# Supplementary material for: Single-Cell Droplet Microfluidic Screening for Antibodies Specifically Binding to Target Cells
Source: Cell Rep. 2018 Feb 20;22(8):2206–15. doi: 10.1016/j.celrep.2018.01.071 (PMC5842027; doi:10.1016/j.celrep.2018.01.071)
Supplement: Document S1. Supplemental Experimental Procedures, Figures S1–S7, and Tables S1–S3 [file mmc1.pdf]

Cell Reports, Volume *22*

Supplemental Information

Single-Cell Droplet Microfluidic Screening for Antibodies Specifically Binding to Target Cells

Nachiket Shembekar, Hongxing Hu, David Eustace, and Christoph A. Merten

Supplemental information

A] Supplemental Figures & Tables

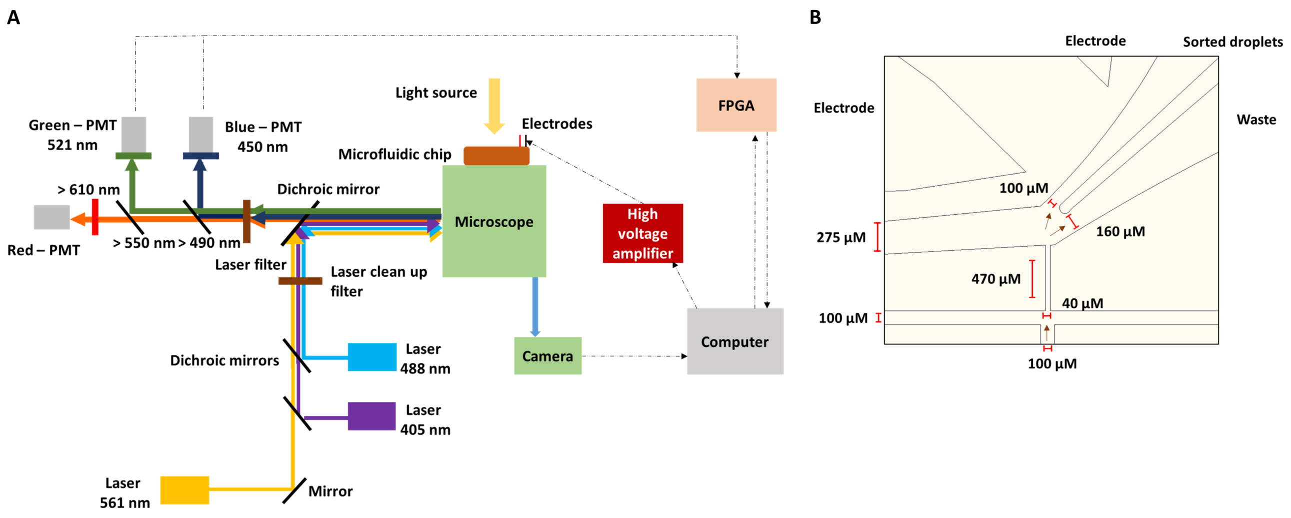

Figure S1: Optical setup and geometry of the sorting junction, Related to Figure 1

[A] The droplets moving in the microfluidic chip were excited with lasers having excitation wavelengths of 405 nm (CTV dye) and 488 nm (Alexa488-goat-anti-mouse IgG antibodies), while the 561 nm laser is an additional optional that was not used in our assays. The emission signals were detected using photomultiplier tubes (PMTs) with a 450 nm band-pass filter (blue), a 521 nm band-pass filter (green), and a 610 nm longpass filter (red). Droplet sorting signals were processed using a LabVIEW software program running on a FPGA card triggering a high voltage amplifier. Imaging was performed using an inverted microscope equipped with a high speed camera. [B] Dimensions of the sorting junction of the microfluidic device used for droplet sorting [Fig. 1B (ii)]. Brown arrows represent the direction in which the droplets move in the sorting device.

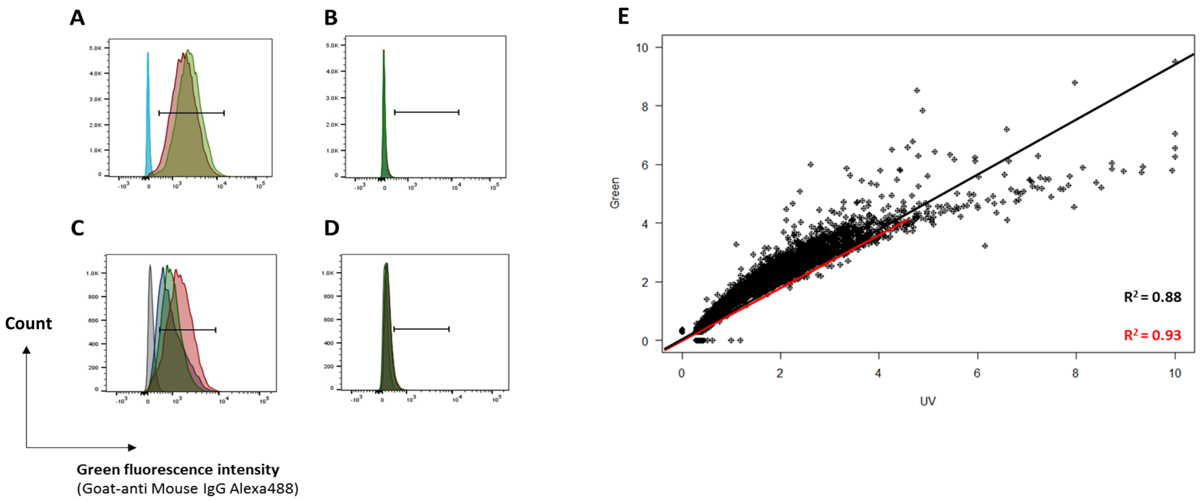

Figure S2: Flow cytometric detection of OKT 9 antibody binding and correlation of normalized signal intensities, Related to Figure 2 and 3

K562 cells were treated with 1:100 (red) and 1:500 (green) dilutions of culture supernatant of OKT 9 [A] or H25B10 [B] cells. The OKT 9 cell secreted antibodies [A] showed significant binding to K562 cells; whereas H25B10 cell secreted antibodies [B] failed to show any binding, as compared to the control (cyan). Further, K562 cells were probed with recombinant OKT 9 [C] or H25B10 [D] antibodies at 50 (blue), 200 (red) and 800 ng/ml (green) concentrations. The OKT 9 antibodies [C] showed binding to the K562 cells whereas H25B10 antibodies [D] did not show any binding, as compared to the control sample (grey). Due to competition for limited secondary antibody between antigen-bound and excessive soluble primary antibody, the OKT 9 binding to K562 cells [C] at 800 ng/ml (green) showed a slightly weaker signal as compared to 200 ng/ml (red). [E] The analysis of significance of correlation between normalized green vs. blue intensity (Fig. 2) showed that >80% of the population shows significant correlation with an R^2-value of 0.93 up to a cut-off value of 4 (shown in red); as compared to an R^2-value of 0.88 (shown in black) for the entire population.

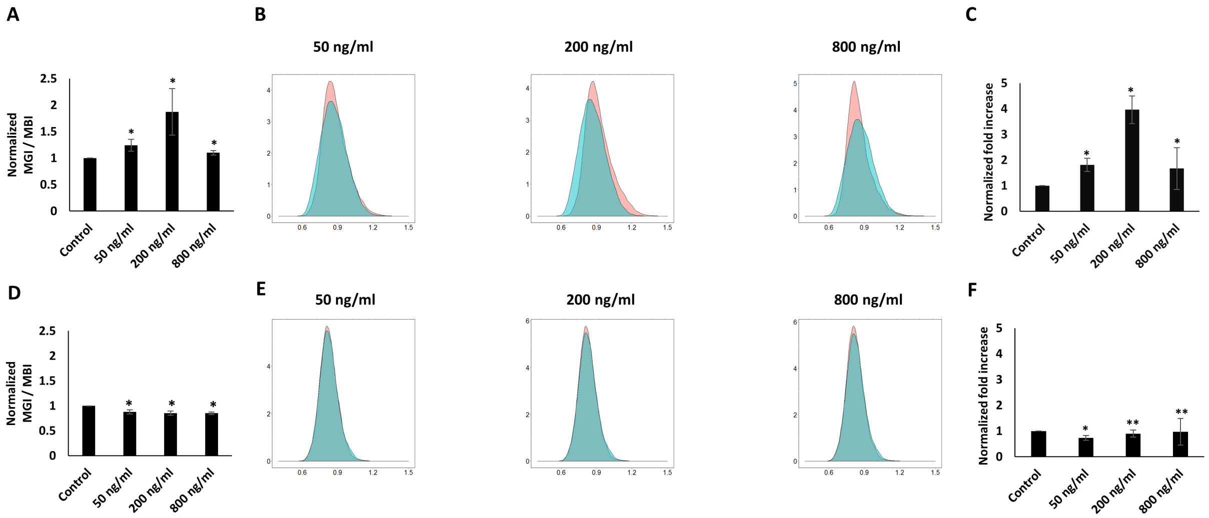

Figure S3: Comparative analysis of relative mean and individual fluorescence intensities from recombinant antibody binding droplet data, Related to Figure 3

To compare the relative increase in fluorescence intensities after recombinant [A] OKT 9 antibody binding on the target cell in droplets, the normalized ratio of Mean Green fluorescence Intensity (MGI) against Mean Blue fluorescence Intensity (MBI) was plotted. It was observed that the [A] OKT 9 antibody binding to target cells lead to relatively higher green fluorescence intensity for 50 – 800 ng/ml antibody as compared to the control (absence of primary antibody). [B] To analyse the robustness of the antibody binding assay, fluorescence intensities after recombinant [B] OKT 9 binding on the K562 cells in droplets were plotted as the ratio of green fluorescence intensity against blue fluorescence intensity for each individual data point shown in Fig. 3A (40,000 – 60,000 peaks in total). [C] Fold increase in the number of data points with a green / blue ratio above 1.1 for OKT 9 over the control (normalized to 1). [D] In contrast, the ratio of MGI / MBI for H25B10 antibody even showed slightly decreased green fluorescence intensity for 50 – 800 ng/ml antibody concentration as compared to the control, potentially indicating a higher level of unspecific binding of the secondary antibody than that of the H25B10 antibody. [E] Similarly, fluorescence intensities after recombinant H25B10 binding on the K562 cells in droplets were plotted as the ratio of green fluorescence intensity against blue fluorescence Intensity for each individual data point shown in Fig. 3B and [F] fold increase in the number of data points with a green / blue ratio above 1.1. Averaged data of 3 independent experiments is shown. The values have been expressed as Mean $\pm$ SD. $*p < 0.05$, $**p > 0.05$ as compared to the control.

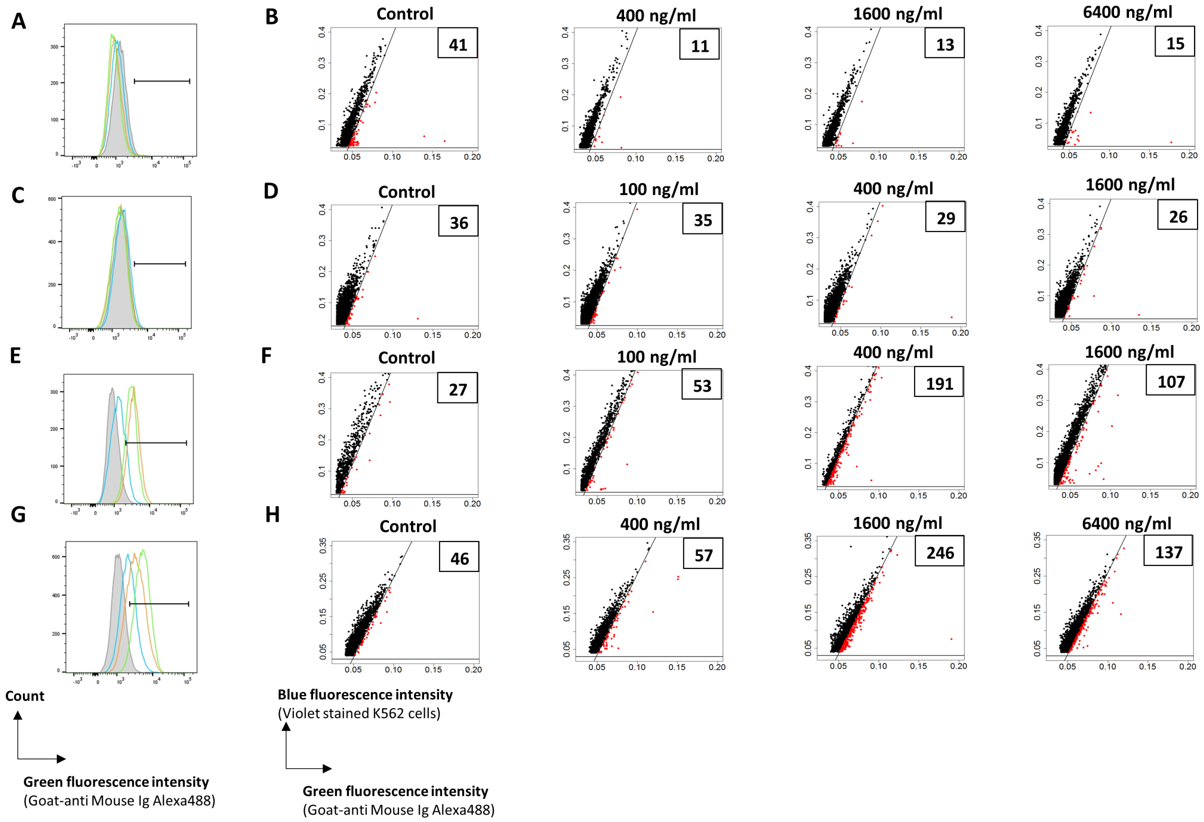

Figure S4: Flow cytometric and droplet based analysis of CD3, MUC1, CD55 and CD59 antibody binding on K562 cell surface, Related to Figure 3

K562 cells treated with recombinant purified CD3 antibody (400-1600-6400 ng/ml; [A] Cyan-Orange-Green, respectively) neither showed any specific antibody binding over the control (filled grey) in flow-cytometric analysis [A] nor in droplets [B] based analysis of fluorescent peaks. Similarly, the K562 cells treated with recombinant purified MUC1 antibody (100-400-1600 ng/ml; [C] Cyan-Orange-Green, respectively) neither showed any specific antibody binding over the control (filled grey) in flow-cytometric analysis [C] nor in droplet [D]. K562 cells treated with recombinant purified CD55 antibody (100-400-1600 ng/ml; Cyan-Orange-Green, respectively) [E] showed significant antibody binding over the control (filled grey) in flow-cytometric analysis. [F] Similar analysis of fluorescent peaks in a droplet based system using CD55 antibody showed significant antibody binding events (53-191-107), over the control sample (27) that did not contain any primary antibody. K562 cells treated with recombinant purified CD59 antibody (400-1600-6400 ng/ml; Cyan-Orange-Green, respectively) [G] showed significant antibody binding over the control (filled grey) in flow-cytometric analysis. [H] Similarly, analysis of fluorescent peaks in a droplet based system using CD59 antibody showed significant antibody binding events (57-246-137), over the control sample (46) that did not contain any primary antibody. Fluorescence peak intensities from 20,000 droplets have been plotted along with horizontal and diagonal lines mimicking a sorting gate [B, D, F and H]. Peaks in the sorting gate have been shown in red color as well as represented in numbers inside the box.

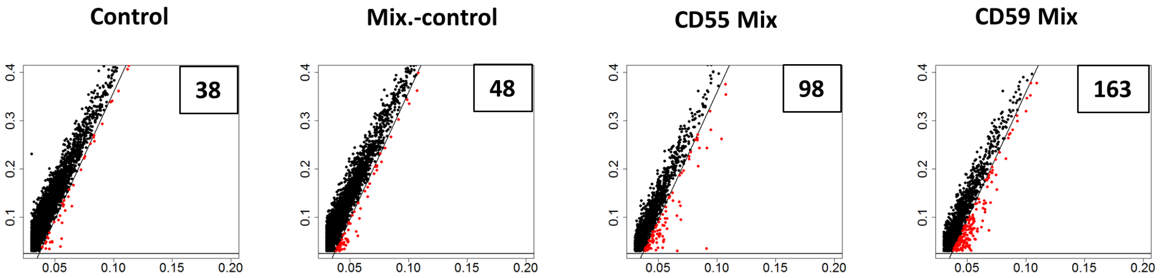

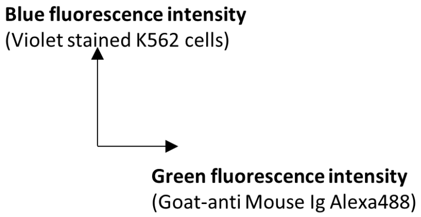

	Control	Mix. –Control	CD55 Mix	CD59 Mix
CD55			√	
CD59				√
H25B10		√	√	√
CD3		√	√	√
MUC1		√	√	√
Anti-Mouse Ig Alexa488	√	√	√	√

Figure S5: Droplet based analysis of CD55 and CD59 antibody binding on K562 cell surface in presence of non-specific antibodies, Related to Figure 4

CTV stained K562 cells along with anti-mouse-Ig-Alexa488 were probed in the droplets either with plain medium (Control) or non-specific antibody mixtures (Mix.-control) or antibody mixtures containing either CD55 or CD59 antibody (CD55 Mix or CD59 Mix) (1600 ng/ml) (see table for details; specific binders are highlighted in green and unspecific antibodies are highlighted in red). The fluorescence peak data obtained from the droplets (20,000 peaks) was plotted as green vs. blue fluorescence intensity. The diagonal and horizontal lines have been drawn so as to mimic a sorting gate to sort droplets showing relatively higher green fluorescence intensity, also represented in red colour and numbered in the box. The presence of CD55 or CD59 antibody even in a mixture of different non-specific antibodies resulted in significantly increased numbers of peaks with relatively higher green fluorescence intensity over the control samples.

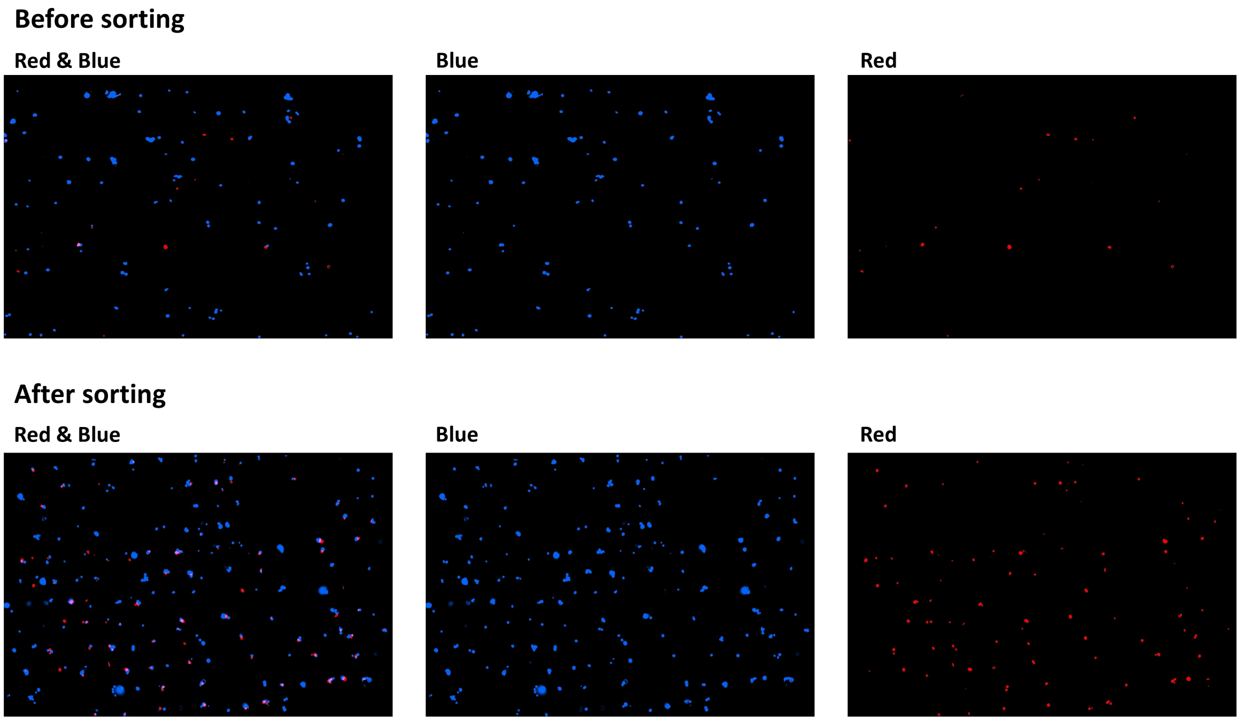

Figure S6: Analysis of a larger population of droplets before and after sorting, Related to Figure 6

To analyse the enrichment of correct droplets after FADS, the droplets before and after sorting were imaged. The K562 cells (blue) and OKT 9 cells (red) were labelled with different dyes and each image is shown in red and blue, blue only and red only channels.

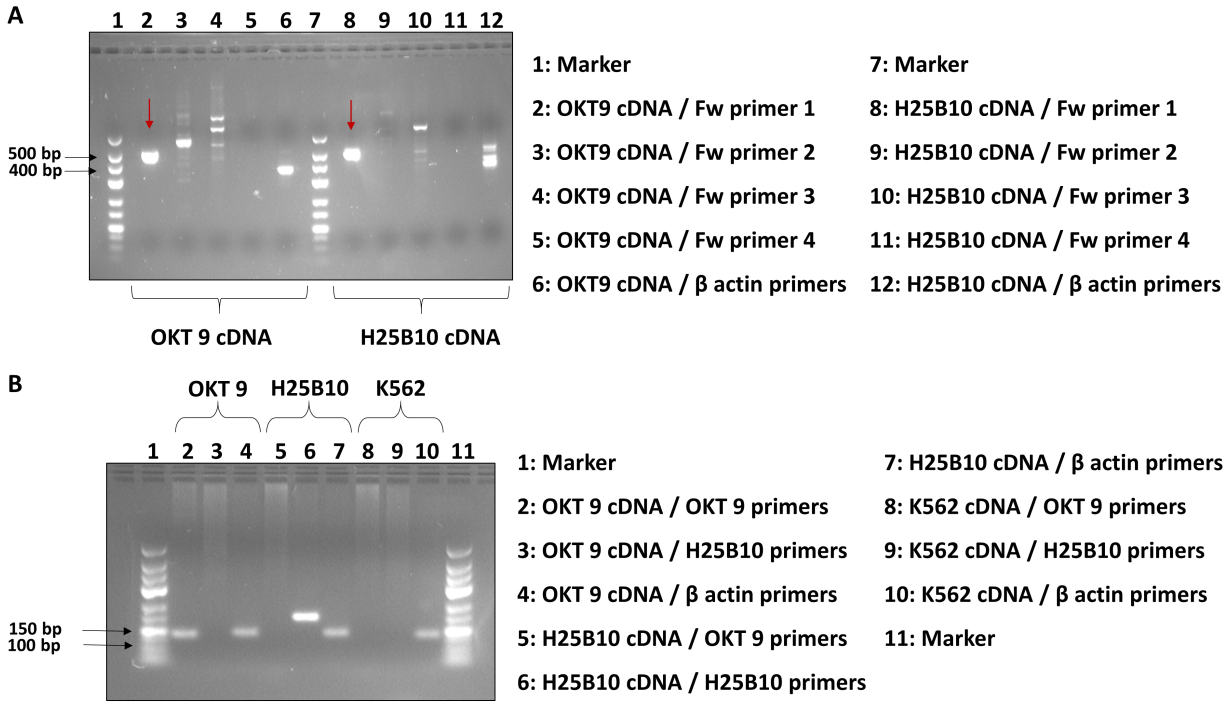

Figure S7: Amplification of OKT 9, H25B10 heavy chain antibody V regions for sequencing and designing of specific Real Time PCR primers, Related to Figure 7

[A] In order to sequence the heavy chain variable (V) region of OKT 9 and H25B10 antibodies, the cDNA from respective hybridoma cells were amplified with universal PCR primers; each in combination with 4 forward (Fw) and 1 reverse primers to amplify the unknown V regions. The amplified products were resolved on 2% agarose gel and products with appropriate size (~450 bp), also shown by red arrows, were further sent for sequencing. Beta-actin primers were used as a non-quantitative internal positive control for the PCR. [B] Real time PCR primers were designed to specifically amplify a sequence (~ 100 bp) within the heavy chain V region of OKT 9 (2, 3) and H25B10 (5, 6) antibodies. In addition, β-actin primers were also designed to amplify beta actin gene (~100 bp) in OKT 9 (4), H25B10 (7) and K562 (10) cell lines, for normalization of real time PCR data. All the PCR products were resolved on 2% agarose gel. All the primers were designed to have similar Tm (48^0 C) and amplification efficiency.

Table S1: Primers for amplification of OKT9 and H25B10 antibody heavy chain V region, Related to Figure 7

No.	Primer	Sequence
1.	Mouse Antibody Heavy chain reverse	5 ′- AGGTCTAGAAYCTCCACACACAGGRRCCAGTGGATAGAC - 3′
2.	Mouse Antibody Heavy chain forward 1	5′- GGGGATATCCACCATGGRATGSAGCTGKGTMATSCTCTT- 3′
3.	Mouse Antibody Heavy chain forward 2	5′-GGGGATATCCACCATGRACTTCGGGYTGAGCTKGGTTTT-3′
4.	Mouse Antibody Heavy chain forward 3	5′-GGGGATATCCACCATGGCTGTCTTGGGGCTGCTCTTCT-3′
5.	Mouse Antibody Heavy chain forward 4	5′-GGGGATATCCACCATGGRCAGRCTTACWTYY-3′

Table S2: Real time PCR primers for specific amplification of Beta actin, OKT 9 and H25B10 antibody V regions, Related to Figure 7

No.	Primer	Sequence
1.	OKT 9 forward	5' – CTGTCAGGAACTGCAGGT – 3'
2.	OKT 9 reverse	5' – GGAAATATTCATTGAAGT – 3'
3.	H25B10 forward	5' – CCTAGCAACGGTCGTTCC – 3'
4.	H25B10 reverse	5' – AAGGTTACCTACTCTTGC – 3'
5.	Beta-actin forward	5' – TGTATGAAGGCTTTGGTCTCC – 3'
6.	Beta-actin reverse	5' – TGTGCACTTTTATTGGTCTCAAG – 3'

Table S3: Real time PCR standard curve for analysis of enrichment of OKT 9 cells, Related to Figure 7

No. of K562 Cells	No. of OKT 9 cells	No. of H25B10 cells	Ct value for OKT 9 amplification	Ct value for H25B10 amplification	Ratio of Ct values (OKT 9 / H25B10)	% OKT 9 cells enrichment
50	50	-	26.05	30.2	0.86	100
50	40	10	26.88	30.1	0.89	80
50	25	25	30.24	28.37	1.10	50
50	10	40	31.93	27.45	1.20	20
50	-	50	32.99	26.58	1.24	0

B] Supplemental experimental procedures

Cell lines, antibodies and dyes

The K562 (ATCC® CCL-243™), OKT 9 hybridoma (ATCC® CRL-8021™) and H25B10 hybridoma (ATCC® CRL-8017A™) cell lines were purchased from ATCC. The K562 cells were cultured in Iscove's Modified Dulbecco's Medium (IMDM) (ATCC® 30-2005™) with 10% FBS (Gibco); whereas OKT 9 and H25B10 hybridoma cells were cultured in IMDM with 20% FBS. Recombinant OKT9 mouse monoclonal antibody (Anti-human CD71, Transferrin receptor) was purchased from affymetrix eBioscience. Recombinant H25B10 monoclonal antibody (Mouse anti-Hepatitis B virus surface antigen) was procured from Merck Millipore. Mouse anti-human-CD55 antibody (referred to as "CD55"), mouse anti-human-CD59 antibody (referred to as "CD59") and mouse anti-human-CD3 antibody (referred to as "CD3") were purchased from Biolegend, USA. Mouse-anti-human MUC1 antibody (referred to as "MUC1") was obtained from Abcam. Goat-anti-mouse IgG Alexa fluor 488 conjugated antibody, CellTrace Violet and CellTrace Far Red dyes were purchased from Thermo Fisher scientific.

Design and fabrication of microfluidic devices

All the microfluidic devices were designed in AutoCAD and fabricated using soft lithography as described previously (Hu et al., 2015). Briefly, silicon wafers (Silicon Materials) were coated with SU-8 photoresist (Microchem) and patterned by UV-illumination through high resolution lithography masks (Selba). Microfluidic chips were prepared by pouring a mixture of 9:1 (w/w) Polydimethylsiloxane (PDMS) elastomer (Sylgard 184 polymer base; Dow Corning) and curing agent (Dow Corning) over the molds which were then cured overnight at 65^0 C. Polymerized PDMS was peeled off from the molds and inlets for tubings and electrodes were punched by biopsy punches (Acuderm inc.). Subsequently the PDMS-devices were plasma bonded onto microscopy glass slides (Thermo Fisher Scientific Inc.; chips for droplet production and capture) or onto ITO glass (Delta Technologies LTD; chips for sorting). Finally, the channels were made hydrophobic by treatment with Aquapel.

Analysis of fluorescence peak data

The fluorescence peak data obtained from PMT was analysed and plotted using the R-software.

Amplification and sequencing of OKT 9 and H25B10 antibody V region

Total RNA was isolated from OKT 9 and H25B10 hybridoma cells using RNeasy Mini kit (Qiagen). The RNA was then converted to cDNA using Superscript III one step RT PCR with platinum Hi-Fi Taq (Thermo Fisher) employing combination of specific reverse and forward primers for heavy chain V region [Table S1] (Morrison, 2002). The PCR products were resolved on 2 % agarose (Sigma) gel. The products with appropriate size (~450 bp) were eluted out from the gel and sent for sequencing (GATC Biotech).

Re-cultivation of cells and second round of FADS

After the first round of droplet sorting, positively sorted droplets (500 – 1,000) were broken and cells were recovered as described earlier. The recovered cells were then cultured in a 96-well plate in medium containing equal amounts of fresh IMDM-20% FBS and (OKT 9 or H25B10-) conditioned medium for 2 weeks. Subsequently, cells were gradually transferred to 6-well plates and finally to T25 culture flasks. The second round of FADS was performed as described earlier, using equal amounts of K562 cells and re-cultivated hybridoma cells along with the fluorescently labelled goat-anti-Mouse Ig-Alexa488. After the sorting, the cells were again recovered from the sorted droplets and processed for real time PCR analysis, as described earlier.

Supplemental References

HU, H., EUSTACE, D. & MERTEN, C. A. 2015. Efficient cell pairing in droplets using dual-color sorting. *Lab Chip,* 15**,** 3989-93.

MORRISON, S. L. 2002. Cloning, expression, and modification of antibody V regions. *Curr Protoc Immunol,* Chapter 2**,** Unit 2 12.
