# Supplementary material for: Single-Cell Droplet Microfluidic Screening for Antibodies Specifically Binding to Target Cells
Source: Cell Rep. 2018 Feb 20;22(8):2206–15. doi: 10.1016/j.celrep.2018.01.071 (PMC5842027; doi:10.1016/j.celrep.2018.01.071)
Supplement: Document S2. Article plus Supplemental Information [file mmc4.pdf]

Cell Reports

Resource

Single-Cell Droplet Microfluidic Screening for Antibodies Specifically Binding to Target Cells

Graphical Abstract

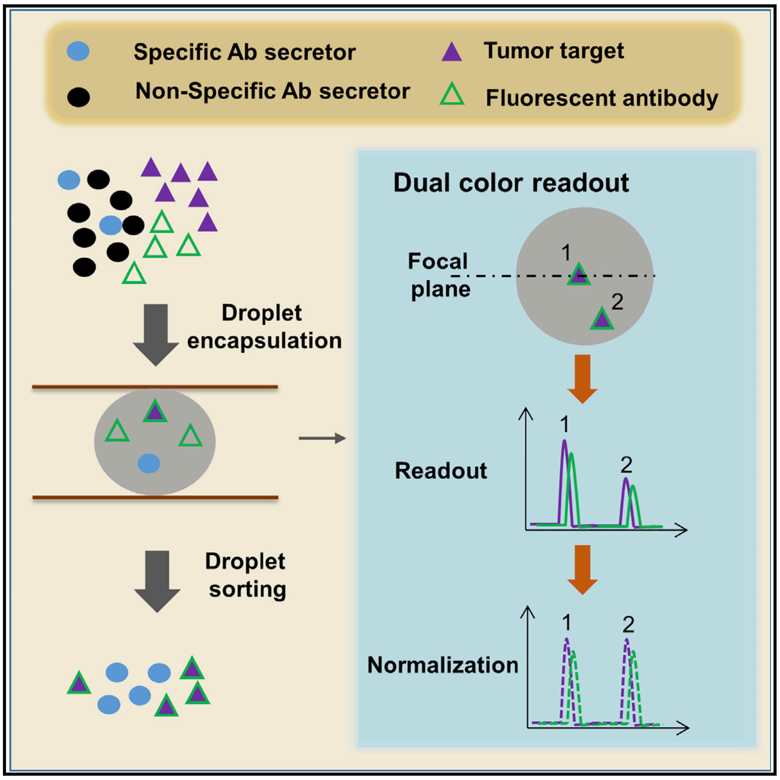

Highlights

- Binding assay with co-encapsulation of hybridoma and target cell in droplets
- Signal normalization allows quantitative detection of Ab binding without focusing
- Droplet sorting for antibody binding shows enrichment of specific hybridoma cells
- 33 fg of antibody can be detected and up to 80,000 clones can be screened

Authors

Nachiket Shembekar, Hongxing Hu, David Eustace, Christoph A. Merten

Correspondence

merten@embl.de

In Brief

Shembekar et al. develop a droplet microfluidics-based antibody binding assay involving co-encapsulation of antibody secreting cells and target tumor cells in the same droplet. This platform enables high-throughput antibody screening on the single-cell level to enrich specific antibodies binding to cell-surface receptors.

Shembekar et al., 2018, Cell Reports *22*, 2206–2215
February 20, 2018 © 2018 The Authors.
https://doi.org/10.1016/j.celrep.2018.01.071

CellPress

OPEN
ACCESS
CellPress

Single-Cell Droplet Microfluidic Screening for Antibodies Specifically Binding to Target Cells

Nachiket Shembekar,[1] Hongxing Hu,[1] David Eustace,[1] and Christoph A. Merten[1,2,*]
[1]European Molecular Biology Laboratory (EMBL), Genome Biology Unit, Meyerhofstrasse 1, Heidelberg, Germany
[2]Lead Contact
*Correspondence: merten@embl.de
https://doi.org/10.1016/j.celrep.2018.01.071

SUMMARY

Monoclonal antibodies are a main player in modern drug discovery. Many antibody screening formats exist, each with specific advantages and limitations. Nonetheless, it remains challenging to screen antibodies for the binding of cell-surface receptors (the most important class of all drug targets) or for the binding to target cells rather than purified proteins. Here, we present a high-throughput droplet microfluidics approach employing dual-color normalized fluorescence readout to detect antibody binding. This enables us to obtain quantitative data on target cell recognition, using as little as 33 fg of IgG per assay. Starting with an excess of hybridoma cells releasing unspecific antibodies, individual clones secreting specific binders (of target cells co-encapsulated into droplets) could be enriched 220-fold after sorting 80,000 clones in a single experiment. This opens the way for therapeutic antibody discovery, especially since the single-cell approach is in principle also applicable to primary human plasma cells.

INTRODUCTION

Monoclonal antibodies are the biggest class of biopharmaceuticals used clinically against cancer, autoimmune diseases, inflammatory diseases, and several other clinical conditions (Nelson et al., 2010; Scott et al., 2012; Weiner, 2015). It is speculated that the global antibody market is set to reach $125 billion by 2020 (Ecker et al., 2015). More than 50% of the currently marketed therapeutic antibodies are targeted against cell-surface receptors (Reichert, 2012, 2016, 2017). These surface receptor targeting antibodies such as trastuzumab (anti-HER2) and cetuximab (anti-EGFR [Epidermal Growth Factor Receptor]) act by either inducing apoptosis in cells or opsonizing the target cell for destruction or by preventing the receptor-ligand interaction or by interfering with its oligomerization process (Chames et al., 2009). However, despite the major clinical importance of such therapeutics, there are several challenges in isolating antibodies against cell-surface proteins: a major requirement is the availability of a conformationally stable, native, and pure receptor molecule as a target antigen. Since the surface-expressed proteins are embedded in the lipid bilayer, their soluble forms are not always conformationally stable (Hutchings et al., 2010). Also, many of these surface molecules are expressed at a low level, and hence there are difficulties in obtaining their purified forms in abundant amounts for screening purposes (Midgett and Madden, 2007). Hence, it is imperative to use whole-cell antigen target for antibody screening.

Technologies to screen antibodies for the binding of cell-surface receptors face certain limitations: conventional hybridoma screens typically do not allow to assay more than just a few thousand clones, are cost intense, and take several weeks until completion. Furthermore, microtiter-plate-based formats are dependent on cell proliferation to obtain sufficient amounts of antibodies for screening. Hence, there is very little room to overcome the need for immortalization (correlating with a dramatic loss in diversity) and apply the same assay principles to primary human cells, even though such approaches would have tremendous clinical potential (e.g., screening of plasma cells from disease survivors). Display technique such as phage or ribosome display provide greater immune diversity but require several panning cycles and may involve unnatural pairing of antibody heavy- and light-chain genes. These limitations can be overcome by using droplet microfluidics technology to perform antibody screening (Shembekar et al., 2016). In these systems, tiny aqueous droplets (~660 pL in volume) surrounded by oil serve as independent reaction vessels for individual antibody secreting cells. Hence the technology is in principle also applicable to non-immortalized plasma cells. We have previously used such an approach to assay antibodies directly for their ability to functionally inhibit the catalytic activity of an enzymatic drug target (El Debs et al., 2012). Binding assays in droplets similar to FMAT (fluorescent microvolume assay technology) (Mazutis et al., 2013; Konry et al., 2011) or based on enzyme-labeled antibodies (Joensson et al., 2009; Chaipan et al., 2017) have also been established, but a platform to screen antibodies for the specific recognition of target cells (co-encapsulated into droplets) has not yet been described. Even more important, previous approaches have not been able to demonstrate the enrichment of specific antibody secretors.

Here, we present a microfluidic system that can overcome these limitations, making use of a dual-color signal normalization approach. We demonstrate the benefit of this approach to screen antibodies for the specific binding of cancer target cells in a high-throughput single-cell format, which should have immense clinical potential.

 © 2018 The Authors.
This is an open access article under the CC BY license (http://creativecommons.org/licenses/by/4.0/).

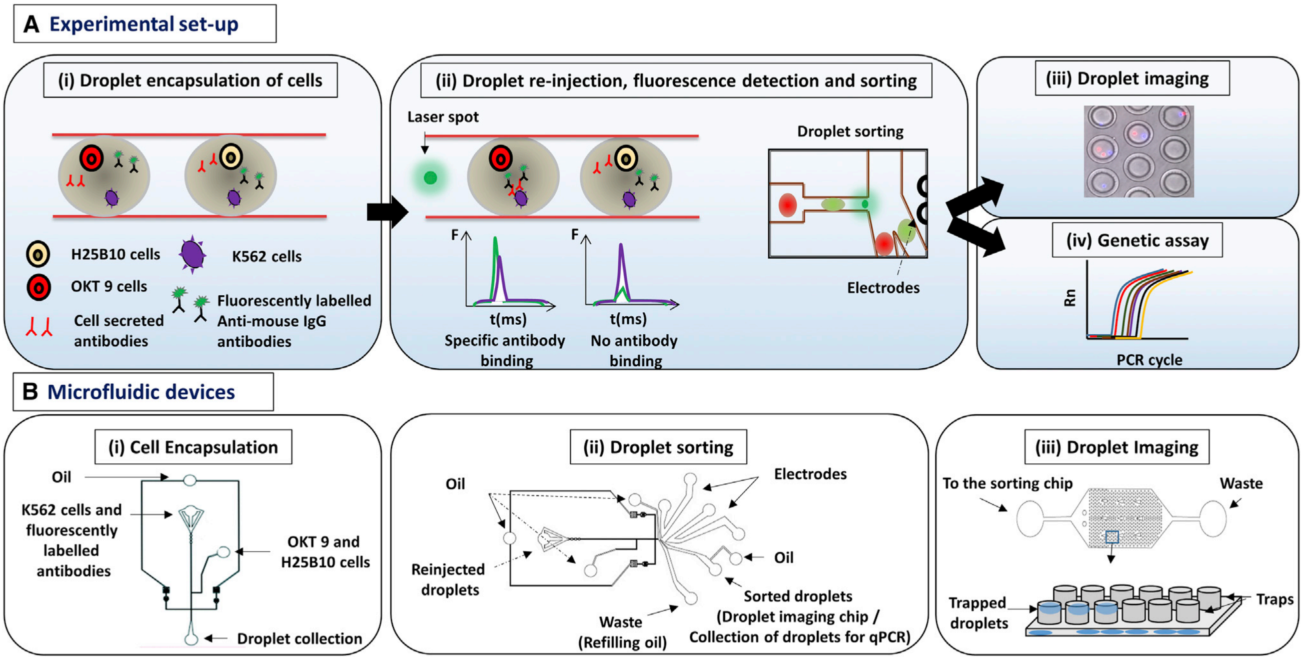

Figure 1. Schematic Overview of Antibody Binding Assay in Droplets

(A) (i) K562 target cells were co-encapsulated in droplets with OKT 9 and H25B10 hybridoma cells, along with fluorescently labeled anti-mouse antibodies. K562 cells were stained with violet stain in all experiments, whereas OKT 9 cells were stained with red dye only for imaging experiments. (ii) The droplets were re-injected into a sorting device and excited with laser. Upon specific binding of OKT 9 secreted antibodies to K562 cell surface, a sharp fluorescence peak was observed. However, non-specific antibodies secreted by H25B10 cells failed to show binding to K562 cells, as a result fluorescence peak was not observed. Based on the fluorescence peak data, droplets were sorted by dielectrophoresis mechanism. (iii) The individual droplets were captured in traps and imaged to determine the cell occupancy before and after droplet sorting thereby revealing the sorting efficiency. (iv) Alternatively, the enriched cell population obtained after antibody binding-based droplet sorting, was processed for a real-time PCR assay to determine the sorting efficiency.

(B) (i) The microfluidic device used for generating ~100-μM aqueous droplets in oil has been shown. The K562 cells and fluorescently labeled anti-mouse antibodies were introduced together, whereas OKT 9 and H25B10 cells were introduced through a different inlet, as indicated by arrows. (ii) The microfluidic device used for droplet sorting has been shown. The functions of various inlets have been indicated by arrows (Hu et al., 2015). (iii) The microfluidic device used for trapping droplets has been shown (Hu et al., 2015). As depicted in the cartoon, inverted traps capture the droplets, which can then be imaged.

RESULTS

Outline of the Antibody Binding Assay in Droplets

Our antibody binding assay is based on the co-encapsulation of an antibody-secreting cell and a target cell into the same microfluidic droplet. As a model system, we chose OKT9 hybridoma cells releasing antibodies binding to the transferrin receptors on leukemic K562 cells (Sutherland et al., 1981). As a negative control, H25B10 hybridoma cells secreting non-related antibodies (whose target Hepatitis B virus Surface Antigen [HBsAg] is not expressed on K562 cells) were used. For microfluidic screening, hybridoma cells were co-encapsulated into droplets of size of about 100 μM, together with K562 target cells and fluorescently labeled goat anti-mouse immunoglobulin G (IgG) Alexa 488 antibody (Figures 1Ai and 1Bi). After generation of the aqueous droplets, the resulting emulsion was incubated off chip for 1–2 hr (to allow for efficient production of antibodies [Abs] inside the droplets) and then re-injected into a sorting chip where the droplets were sequentially assayed by laser spectroscopy (Figures 1Aii, 1Bii, and S1A). If the antibodies secreted by the hybridoma cells bound to the K562 cells, the secondary fluorescently labeled antibodies got co-localized on the target cell resulting in a sharp fluorescent peak, whereas in absence of any specific antibody binding the secondary antibodies remained homogeneously distributed in the droplets and no fluorescence peak was observed (Figure 1Aii). Based on the fluorescence peaks, the droplets were applied to dielectrophoretic sorting (Figures 1Aii, 1Bii, and S1B). The droplet sorting efficiency and the enrichment of specific antibody secreting cells were determined in two ways: (1) The sorted droplets were individually trapped in a microfluidic chip and they were imaged (Hu et al., 2015). By pre-staining the target and specific OKT 9 cells with different dyes, the cell population in the sorted droplets could be analyzed (Figures 1Aiii and 1Biii). (2) Alternatively, the sorted cell population was recovered from the droplets and applied to a real-time PCR assay employing antibody variable (V) region-specific primers for OKT 9 and H25B10 cells (Figure 1Aiv).

Characterization of Model Antibodies by Flow Cytometry

We used a model system closely mimicking an antibody screening process for tumor antigens, with the K562 cell line as a proxy tumor target and OKT 9 or H25B10 cells as proxy

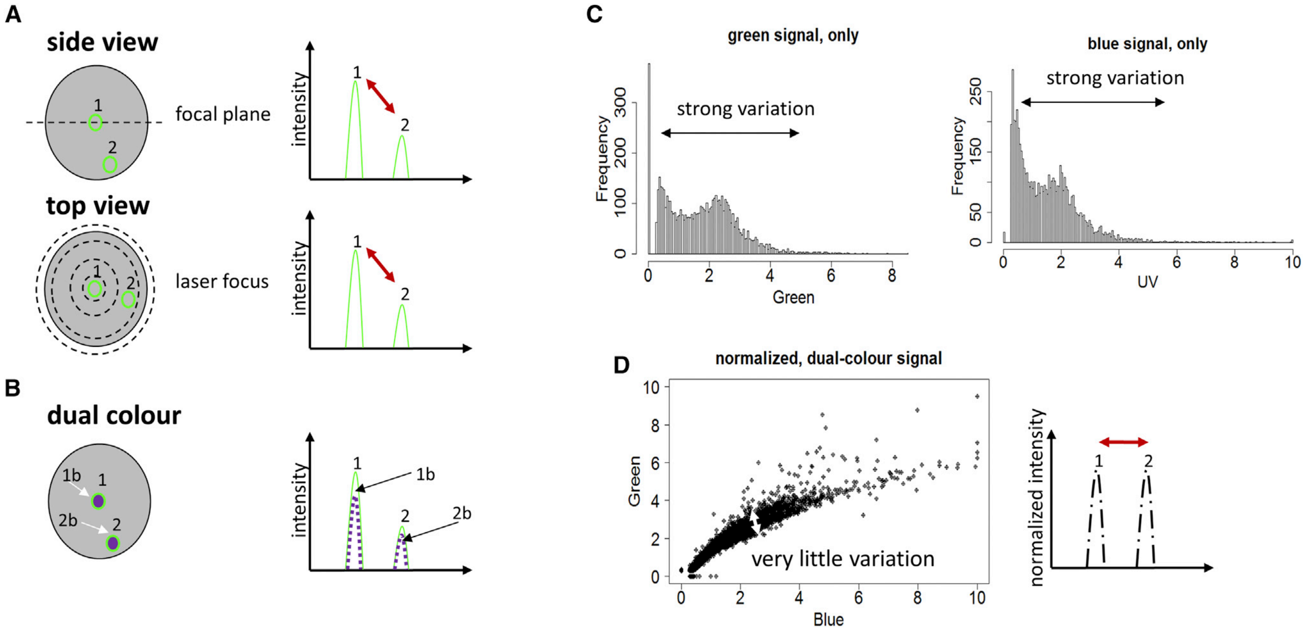

Figure 2. Normalization of the Fluorescence Readout from Droplets

(A) The measured fluorescence signal from a droplet can vary if the particle is outside the focal plane (top left, 1 and 2). In this case, the fluorescence peak (top right) is weaker (top right, 2) as compared to a particle within the focal plane (top right, 1). The same is also true for particles outside the center of the laser focus (bottom left, 1 and 2), which can give rise to a weaker fluorescence signal (bottom right, 1 and 2).

(B) However, this problem can be overcome, by using a second fluorescent color for the readout (e.g., a blue fluorescent dye, indicated by arrows and/or the label "b" in the bottom panel) on the beads. By normalizing the green signal with the blue signal, the variations in the signal can be minimized.

(C) The blue-green beads were encapsulated in the droplets, excited with green and blue laser and the fluorescence signal data from the droplets was recorded. It was observed that the individual green (left) as well blue (right) signals showed significant variation (indicated by arrow). The beads, which were expected to give uniform fluorescence intensities, showed variation possibly due to the variable position of the bead inside the droplet.

(D) When the blue and green signal coming from the droplets were normalized with each other, variation in the signals was significantly reduced. In this way, the fluorescence readout e.g., the number of bound green antibodies on a target can be measured quantitatively and independently of the position of the target inside the droplet.

B cells. In addition, the purified recombinant antibodies secreted by both the hybridomas were also available commercially, which was exploited to obtain quantitative data. Initially, in a flow cytometric analysis, we confirmed that the culture supernatant of the OKT 9 hybridoma cells (cell secreted OKT 9 antibodies) showed antibody binding to K562 cells, whereas culture supernatant of H25B10 hybridoma cells (cell secreted H25B10 antibodies) did not show any specific antibody binding to K562 cells (Figures S2A and S2B). Also, we treated K562 cells with varying concentrations of OKT 9 and H25B10 recombinant antibodies from 50 to 800 ng/mL. To mimic an antibody binding assay in droplets, we carried out this experiment by simultaneous addition of recombinant and fluorescently conjugated antibodies to K562 cells and without performing a washing step in between to remove the primary antibodies. Flow cytometric analysis of K562 cells revealed that the recombinant OKT 9 antibody showed significant binding to K562 cells for concentrations from 50 to 200 ng/mL, whereas antibody binding at a concentration of 800 ng/mL was also observed albeit with reduced fluorescence intensity (Figure S2C). Weaker antibody binding signals for increased antibody concentrations are a well-known phenomenon that has been termed "hook effect" in previous literature (Ryall et al., 1982). It is based on an excess of primary antibody in comparison to the fluorescently conjugated secondary antibody. Performing homogeneous assays without any washing step the cell-surface receptors hence get saturated with free primary antibodies that do not show any fluorescence signal. In theory, this effect could be reduced by increasing the concentration of the fluorescently conjugated secondary antibody in the assay. However, this also results in an increase in the background noise signal and therefore a decreased overall sensitivity. Independently of this, recombinant H25B10 antibody did not show any significant binding to K562 cells (Figure S2D), thus demonstrating the expected specificity of our model system.

A Dual-Color Normalized Fluorescence Readout for Droplet Analysis

Having comprehensive flow cytometry data on our model system, we then set out to repeat the binding assays in a droplet format. However, this posed one inevitable problem: the fluorescence signals of target cells having bound primary and fluorescently labeled secondary antibody strongly varied depending on their position within the droplet. For example, cells can be closer to or further away from the focal plane and the center of the laser spot, resulting in higher or lower fluorescence

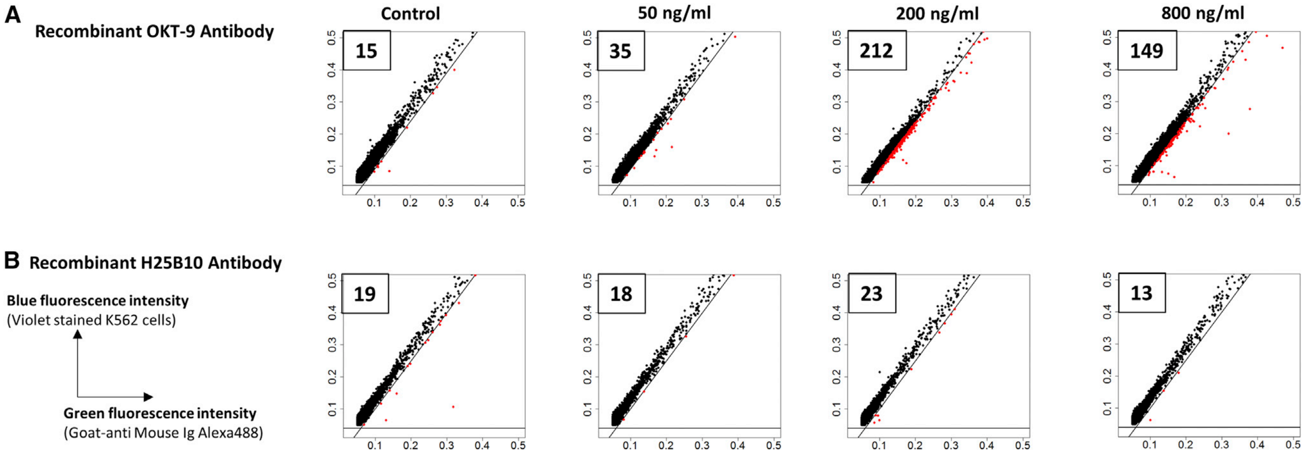

Figure 3. Droplet-Based Analysis of Sensitivity of OKT 9 Antibody Binding on K562 Cell Surface

In order to analyze the sensitivity of antibody binding on cell surface in droplets in quantitative terms, recombinant OKT 9 (A) or H25B10 (B) antibody at 50, 200, and 800 ng/mL were co-encapsulated in droplets along with violet-stained K562 cells and Alexa-488-conjugated anti-mouse Ig antibodies. The fluorescence peak data obtained from the droplets (20,000 peaks) was plotted as green versus blue fluorescence intensity. The diagonal and horizontal lines have been drawn so as to mimic a sorting gate to sort droplets showing relatively higher green fluorescence intensity, also represented in red color.

(A) It was observed that OKT 9 antibody concentrations from 50 to 200 ng/mL showed increasing number of peaks (35–212, indicated in boxes and represented in red color) with relatively higher green fluorescence intensity over the control (15), where OKT 9 antibody was absent. The number of peaks with relatively higher green intensity were reduced at 800 ng/mL (149), due to the saturation of the antigen-antibody interaction.

(B) Increasing concentrations of H25B10 antibody did not show increasing number of peaks with higher green intensity as compared to the control.

intensities (Figure 2A). To analyze this effect in more detail and develop a solution for quantitative measurements, we performed further experiments using 6-μm fluorescent beads emitting in the blue and green channel (Figure 2B). Droplets hosting these beads showed strongly varying signals in the individual blue and green channels (Figure 2C), but when plotting the green intensity against the blue intensity an almost linear correlation was obtained (Figures 2D and S2E). This clearly demonstrated that processing the signals simultaneously in two channels can overcome positional effects: whenever a bead is outside the focal plane not only the assay signal (e.g., green fluorescence) but as well the marker signal (e.g., blue fluorescence) shows a decreased intensity, thus allowing for easy normalization (Figure 2D). To implement a corresponding sorting setup, we developed a LabVIEW program in which normalized sorting gates can be applied. Furthermore, we made the approach applicable to our K562 target cells by staining them additionally with CellTrace Violet (CTV) dye.

Sensitivity of OKT 9 Antibody Binding to K562 Cells in Droplets

Having established the dual-color assay, we were wondering whether it allows quantitative measurement of antibody binding in droplets. To do so, we encapsulated varying concentrations of recombinant purified OKT 9 or H25B10 antibodies into droplets, together with fluorescently labeled anti-mouse IgG antibodies and CTV-labeled K562 cells. The normalized fluorescence peak data obtained from 20,000 droplets was plotted as green- against-blue fluorescence intensity along with reference lines mimicking sorting gates. Increasing the concentration of OKT 9 antibody from 50 to 200 ng/mL resulted in a higher green/blue ratio of the entire population (Figure S3A) and significantly more peaks (212 versus 35) within the sorting gate (Figure 3A). Also, the frequency of peaks with relatively higher green/blue ratio was significantly higher in presence of OKT 9 antibody over the control (Figures S3B and S3C). Interestingly, for even higher concentrations of the OKT 9 antibody (800 ng/mL) the number of peaks within the sorting gate decreased to 149 (Figure 3A). This hook effect had also been observed for the flow cytometry experiments (Figure S2C). The antibody binding event inside a droplet may not happen sequentially with respect to the primary antibody binding to the target and the secondary antibody binding to the primary antibody. Thus, excessive amounts of primary antibody lead to saturation of all cell-surface epitopes and prevent further primary-secondary antibody complexes from binding. In case the concentration of the primary antibody exceeds that of the fluorescently labeled secondary antibody, this inevitably means that less fluorophores bind to the cell surface, resulting in a decreased readout signal. Hence, the concentration of the fluorescently labeled antibody is a critical parameter, which probably has to be optimized for each assay and/or drug target.

Independently of this, our setup showed high specificity: increasing concentrations of the control antibody H25B10 did neither result in a higher number of peaks in the sorting gates nor in a higher green/blue ratio of the entire population (Figures 3B and S3D–S3F). Even when using the optimal concentration of 200 ng/mL IgG, the number of positive events remained at background level (23) (Figure 3B). The presence of primary non-specific antibody such as H25B10 also brought down the background signal as compared to the sample without any primary antibody (control), probably due to competition for limited secondary antibody (Figure S3D).

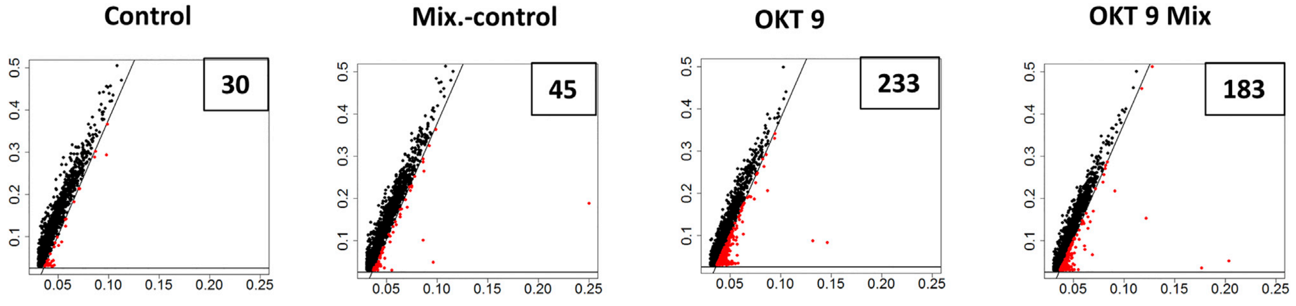

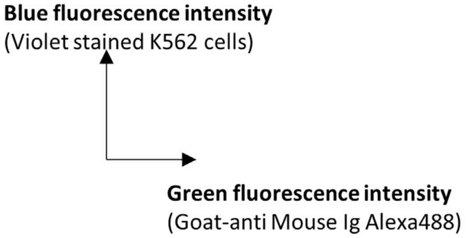

	Control	Mix. –Control	OKT 9	OKT 9 Mix
OKT 9			√	√
H25B10		√		√
CD3		√		√
MUC1		√		√
Anti-Mouse Ig Alexa488	√	√	√	√

Figure 4. Droplet-Based Analysis of OKT 9 Antibody Binding on K562 Cell Surface in Presence of Non-specific Antibodies
CTV-stained K562 cells along with anti-mouse-Ig-Alexa 488 were probed in the droplets either with plain medium (Control) or individual antibodies (OKT 9) or antibody mixtures (OKT 9 Mix) (250 ng/mL) (see table for details; specific binders are highlighted in green and unspecific binders are highlighted in red). The fluorescence peak data obtained from the droplets (20,000 peaks) was plotted as green versus blue fluorescence intensity. The diagonal and horizontal lines have been drawn so as to mimic a sorting gate to sort droplets showing relatively higher green fluorescence intensity, also represented in red color and numbered in the box. The presence of OKT 9 antibody even in a mixture of non-specific antibodies resulted in significantly higher peaks with relatively higher green fluorescence intensity over the control samples.

Specificity of Antibody Binding on K562 Cell Surface in Droplets

Next, we aimed for demonstrating that our approach works in a non-binary system as well, and also for more than just one target protein. In a first step, we addressed potential stickiness of different unspecific antibodies and performed experiments with two additional negative control antibodies, anti-CD3 (hereafter referred to as "CD3") and anti-MUC1 (hereafter referred to as "MUC1"), which are specific for cell-surface receptors not expressed on K562 cells. Flow cytometric as well as droplet-based analyses showed that neither CD3 nor MUC1 antibody showed detectable antibody binding to the K562 cell surface (Figures S4A–S4D). Then, we set up a "Mix.-control" sample containing all non-specific antibodies together (H25B10, CD3, and MUC1) and a positive sample containing OKT 9 in addition to the non-specific antibodies (Figure 4). Analysis of the droplet fluorescence data revealed that the Mix.-control sample did not show any significant background peaks over the control indicating minimal "stickiness" due to the non-specific antibodies (Figure 4). The mixture of antibodies containing OKT 9 antibody (OKT 9 Mix) still showed higher number of data points (183) in the sorting gate, over the Mix.-control sample, indicating specific binding of the OKT 9 antibody to the K562 cells even in presence of various non-specific antibodies (Figure 4). The difference in the number of peaks in the sorting gate due to the OKT 9 Mix as compared to the OKT 9 sample (183 versus 233) (Figure 4) could be attributed to the relative excess of primary antibodies in the OKT 9 Mix sample, which may lead to a competition for the limiting fluorescently labeled secondary antibody.

In a second step, we then set out to demonstrate the general applicability of our system to more than one target protein. For this purpose, we included two further recombinant purified antibodies, anti-CD55 (hereafter referred as "CD55") and anti-CD59 (hereafter referred as "CD59"), and demonstrated specific binding to the K562 cells over a wide range of concentrations in flow cytometric as well as droplet-based analyses (Figures S4E–S4H). Analysis of CD55 and CD59 antibodies in the droplet system revealed higher numbers of antibody binding events in the sorting gate as compared to the control, in a similar manner as observed for the OKT 9 antibody (Figures S4F and S4H). Interestingly, the hook effect was also evident with the higher concentrations of CD55 and CD59 antibodies (Figures S4F and S4H). Nonetheless, the data clearly show that specific binding of antibodies to various surface receptors can be detected in the droplet microfluidic setup.

Similar to the experiments with OKT 9, we also used CD55 and CD59 antibodies to confirm that specific antibody binding can be detected even in presence of all three non-specific antibodies (Figure S5). Flow cytometrically determined approximate binding affinities (Chao et al., 2004), indicated K_{Ds} between 1 and 20 nM for OKT 9, CD55, and CD59 antibodies on K562 cells, demonstrating that our approach is not dependent on antibodies with extraordinary high affinity. Taken together, this clearly shows that our approach is robust and applicable to many different antibodies and targets.

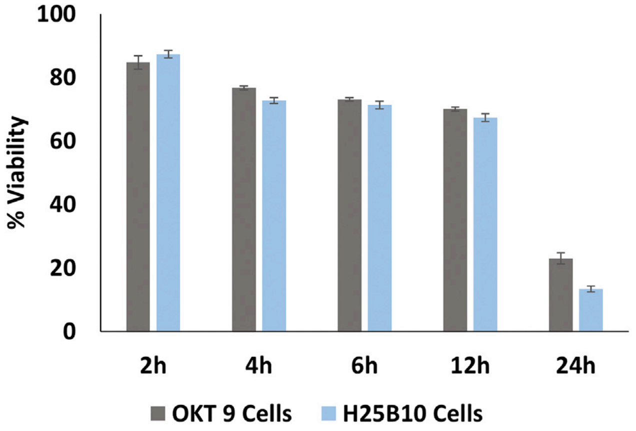

Figure 5. Viability Analysis of OKT 9 and H25B10 Cells after Incubation in Droplets

To assess the viability of OKT 9 and H25B10 cells in droplets, the cells were recovered from the droplets at various time intervals (2, 4, 6, 12, and 24 hr) and stained with a solution of Calcein AM (viable cells, green) and ethidium homodimer (non-viable cells, red). Mean of the percentage of viable cells ± SD from 3 independent experiments is shown.

Droplet Imaging Analysis after FADS Shows Enrichment of K562 and OKT 9 Hybridoma Cells

Subsequent to purely analytical experiments, we aimed for demonstrating specific selection of cells secreting antibodies binding to the leukemic target cells. Before performing droplet sorting experiments, we analyzed the viability of OKT 9 and H25B10 cells in droplets over a period of 24 hr. The cell viability assay was done using viability dyes Calcein AM and Ethidium homodimer, as described previously (Clausell-Tormos et al., 2008). We observed that close to 80% of the cells were viable for typical incubation times of 2–4 hr, as required for sufficient production of antibodies in droplets (El Debs et al., 2012) (Figure 5). We did not examine the viability of K562 cells in the droplets since these cells were anyway fixed with PFA prior to the encapsulation in droplets. For sorting, we started with a 1:10 mixture of OKT 9 and H25B10 cells and encapsulated individual hybridoma cells into droplets, together with CTV-labeled K562 cells and Alexa 488 anti-mouse IgG antibodies. To monitor the sorting enrichment, the positive OKT9 cells were stained with a CellTrace Far Red (CTFR) dye prior to encapsulation. Imaging of the droplets before sorting revealed that about 5%–13% of the samples contained exactly one OKT9 and at least one K562 cell, which is in good agreement with Poisson statistics. FADS (fluorescence activated droplet sorting) was performed by setting up a threshold on normalized fluorescence peak data to select double-positive droplets showing violet and green fluorescence signals. This sorting mode also works in case there are more than 2 target cells in the same droplet, despite the fact that in this case the amount of available antibody is shared by both target cells. However, our data on recombinant antibodies showed that positive events can be detected over a wide range of primary antibody concentrations. The sorting was performed up to a frequency of 40 Hz as shown in Movie S1. The droplets were captured in droplet traps, before and after sorting, and images were taken to analyze the cell population in the droplets. Before sorting, 40%–50% of the droplet population consisted of mainly empty droplets or droplets containing only target K562 cells (blue cells), whereas 4%–10% droplets contained both K562 and OKT 9 cells (blue and red cells) and about 5% contained only OKT 9 cells (red) (Figure 6A). However, upon sorting the droplets containing K562 and OKT 9 cells could be enriched to up to 70% (Figures 6B, 6C, and S6). Importantly, images in the green field revealed that all and only the target K562 cells showed green staining, indicating the localization of the anti-mouse IgG fluorescent antibody (Figure 6B). There was still a population of droplets containing only K562 cells after sorting. These were most likely due to significant amounts of OKT 9 antibodies secreted by the hybridoma cells in the medium, which bound to the K562 cells before or during encapsulation (inside the syringe used to inject the hybridoma cell mixture into the microfluidic chip). We tried minimizing this effect by cooling down the hybridoma cells, before and even during the encapsulation process. However, it seemed impossible to completely overcome this phenomenon, leading to some degree of sorting impurities. Nonetheless, the majority of selected cells were true positives, showing that this is not a major limitation.

Real-Time PCR Analysis upon FADS Shows Enrichment of Specific OKT 9 Cells

When screening diverse libraries of antibody secreting cells, the V regions of the antibody encoding genes need to be amplified after sorting in order to isolate and express selected binders. To demonstrate this and determine the sorting efficiency in a second independent assay, we also performed real-time PCR assays, using primers specific for V regions of OKT 9 and H25B10 cells. In order to sequence the heavy-chain V regions of the OKT 9 and H25B10 antibodies, the V regions were initially amplified with universal primers (Figure S7A; Table S1). The products with appropriate lengths (~450 bp) were sent for Sanger sequencing. After receiving the sequences, specific real-time PCR primers amplifying a unique stretch (~100 bp) in the heavy-chain V regions of OKT 9 and H25B10 were designed (Figure S7B; Table S2). In addition, we decided to use β-actin gene marker for normalization of the amount of input DNA before and after sorting (Figure S7B; Table S2). To mimic an antibody screening assay, OKT 9 cells were spiked into an excess of H25B10 cells at different ratios (1:20, 1:100, 1:400). After performing FADS for antibody binding on K562 cells, the droplets before and after sorting were broken; cells were recovered, and RT-PCR assays were carried out. Generally, a complete sorting experiment took 7–8 hr, and typically at least 100 positively sorted hybridoma cells could be recovered at the lowest (1:400) spike in ratio. For obtaining quantitative data on OKT 9 cell enrichment after sorting, a standard curve was prepared by mixing OKT 9 and H25B10 cells in various ratios in bulk and comparing the ratio of their cycle threshold (Ct) values (Figure 7A; Table S3). Real-time PCR analysis of the sorted samples then confirmed that the OKT 9 cells could be enriched ~14.2-fold for the 1:20 starting ratio and up to ~220-fold for the 1:400 spike-in ratio (Figure 7B). This means that despite the complex setup of the assay (encapsulation of three different cell types in

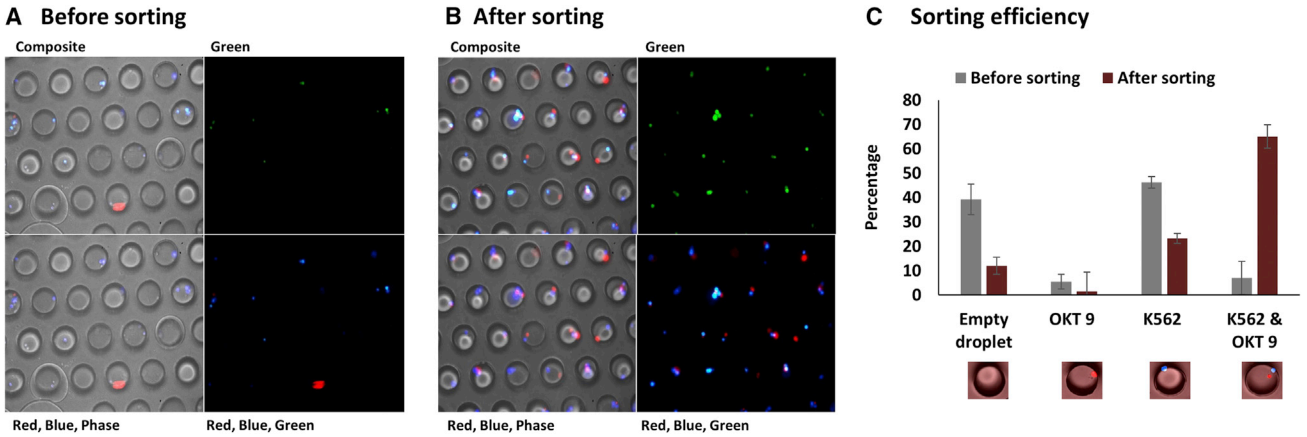

Figure 6. Image Analysis of Droplets before and after Sorting for the Binding of Antibodies to the K562 Cell Surface

Droplets containing K562 cells (Blue), OKT 9 cells (Red), H25B10 cells (Unstained) along with the Alexa-488-labeled anti-mouse IgG antibodies were sorted using FADS to select for specific antibodies binding to K562 cells.

(A and B) Representative images of the droplets captured in traps before (A) and after (B) sorting. Each image is shown as composite, green, red-blue-phase, and red-blue-green. The images also reveal the "green" staining of K562 cells indicating localization of the fluorescent anti-mouse IgG antibodies.

(C) The percentage of droplet population before and after sorting was calculated from 3 different experiments with examination of at least 3 different fields of view, which revealed enrichment of droplets containing OKT 9 and K562 cells from 4%–10% to up to 70%.

Mean of the percent droplet population ± SD is shown.

total; monitoring binding without any washing step) the majority of hits are true positives.

Next, we asked whether the sorted population of hybridoma cells from one FADS round can be amplified and subsequently be used for another round of sorting possibly leading to higher enrichment of specific Ab secretors. We recovered the hybridoma cells from positively sorted droplets of three different 1:400 (OKT 9: H25B10) spike-in experiments and cultured them for about 3–4 weeks to obtain sufficient numbers of cells for another sorting round. The ability to recover and amplify sorted cells once again confirmed that all microfluidic manipulation steps have no major detrimental effect on cell viability. Another round of FADS using these recultivated cells, followed by real-time PCR analysis of sorted cells showed further enrichment of OKT 9 cells by up to ~300-fold (~75%) (Figure 7B). We also demonstrated the semi-automated handling of single cells (e.g., recultivation and sequencing) as required for downstream characterization of individual clones selected from diverse libraries. For this purpose, we immersed a fixed tubing connected to a syringe pump into a dish with selected cells. By moving the microscope stage and aspirating/releasing predefined volumes (in our case <1 μL) single cells can be reliably transferred between different dishes and tubes (Movie S2). Taken together, our platform enables to screen hybridoma cells, over multiple rounds of culture and re-sorting, and isolate specific Ab secretors from a large excess of cells secreting unspecific antibodies.

DISCUSSION

Here, we present a microfluidics platform for the selection of antibodies binding to cell-surface receptors. In contrast to previous studies (Mazutis et al., 2013), our platform not only allows to qualitatively sort for antibody expression, but also specific antibody binding, in a quantitative way, and to native cell-surface receptors. A key element of our approach was the development of a normalization strategy for all fluorescence signals, allowing quantitative fluorescence measurements independently of the position of the target cell within the droplet. Compared to another very recently described approach making use of magnetic fields to align magnetic particles in the focal plane of a droplet (Eyer et al., 2017), our strategy is also applicable to target cells rather than just purified antigens on beads. Furthermore, the current study demonstrates successfully the selection of specific binders from an excess of control antibodies.

Our data suggest that antibody amounts as little as 33 fg (concentration in the droplets × droplet volume = 50 ng/mL × 660 pL; Figure 3A) are sufficient to carry out high-throughput assays. In a previous study, we showed that individual hybridoma cells encapsulated into droplets of the same size can generate antibody concentrations of approximately 20 μg/mL after 6 hr of incubation (El Debs et al., 2012). Assuming a linear rate of antibody expression, this corresponds to a secretion rate of 3.66×10^5 fM/min. So, theoretically, an antibody concentration of 50 ng/mL (corresponding to our detection limit for the OKT 9 antibody) can be achieved in as little as 1 min. However, the hybridoma cells are cooled down before encapsulation into the droplets, which probably leads to delayed maximal secretion rates. Therefore, incubation times of approximately 2 hr for 100-μM droplets (as used here) or ~30 min for 40-μM droplets (Eyer et al., 2017) seem to be optimal for single-cell binding assays.

We typically processed more than one million droplets per screen, corresponding to at least 80,000 droplets hosting an antibody expressing cell and a target cell (for the 1:400 spike in ratio). This hence corresponds to the number of different clones that could be screened and enriched ~220-fold in a

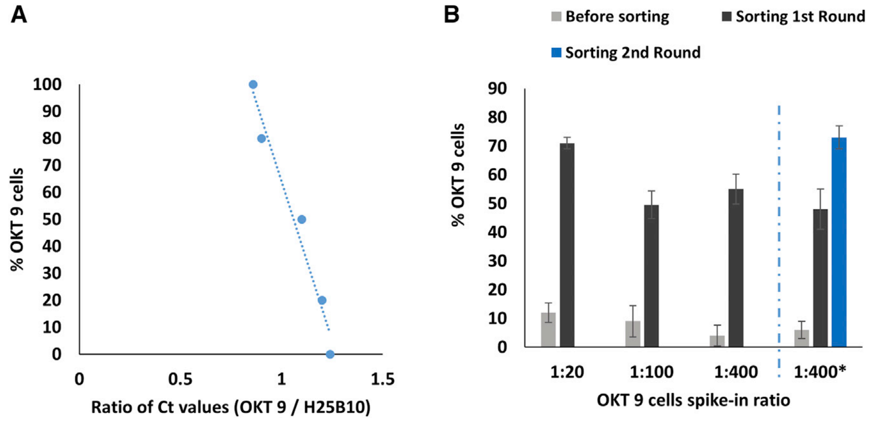

Figure 7. Analysis of Droplet Sorting and Enrichment of OKT 9 Cells by Real-Time PCR

(A) For analyzing the OKT 9 cell enrichment in quantitative terms by real-time PCR, a standard curve was prepared by mixing cells in varying ratios and then comparing their Ct values (Table S3).

(B) Analysis of sorted cell population by real-time PCR showed that the 1:20, 1:100, and 1:400 spike-in ratio for OKT 9 cells before sorting could be enriched to 71%, 49.5%, and 55%, respectively, after FADS. In three separate experiments at 1:400 spike-in ratio (1:400*, separated by blue dotted line), the sorted hybridoma cells were recovered from the droplets, cultured and used for another round of sorting. From an average enrichment of 48% of OKT 9 cells in the first round of sorting, the enrichment could be increased to up to 75% in the second round of sorting.

Mean of the percent of OKT 9 cells from 3 independent experiments ± SD is shown.

single experiment. Compared to existing hybridoma screening approaches (Staudt et al., 2014), droplet-based microfluidics offers dramatic improvement in terms of throughput (1–2 orders of magnitudes more cells), ease, and cost savings. Given that we obtained enrichment rates of up to 220-fold in a single sorting experiment, autofluorescence from cells does not seem to be a major limiting factor for the methodology. However, in case this turns out to be a significant problem for a particular assay, differently labeled antibodies (e.g., blue or red fluorophores) could be used. Cell viability does not seem to be a limiting factor either, given that the target cells in our assays are anyway fixed, and hybridoma cells only have to be viable until sufficient amounts of antibodies are produced within the droplets (antibody sequences can be recovered from dead cells, too, even though our data indicate rather high percentages of viable cells after the sorting). In principle, the sorting rate can be increased further by exploiting smaller droplets, as shown by us previously (Chaipan et al., 2017): using droplet sizes of ~30 μm in diameter, we obtained approximately 10-fold increased sorting rates over periods of up to 40 hr. However, this study did not involve viable cells but rather viral particles. We believe that assays based on the co-encapsulation of two different cells require large droplets as used in this study, even if this limits the maximal sorting rate.

Our screening approach is not dependent on any cell proliferation and hence generally compatible with the screening of primary plasma cells. This is also supported by the fact that antibody concentrations as little as 50 ng/mL are fully sufficient for our approach, while yields up to 250 ng/mL are typically obtained from human plasma cells (Huang et al., 2013). We believe the screening of human material should be of particular interest for the discovery of tumor-specific antibodies: assays as described here could be carried out using plasma and tumor cells from the same patient. Based on the counter selection against self-recognition in the human immune system, the vast majority of all detected binding events should be tumor specific. T cell leukemia is probably most suited for a first case study, given that for this disease the tumor cells are easily accessible. Having this in mind, we used leukemic cells as a model system and provide data demonstrating the general feasibility of corresponding droplet assays: using spike in ratios down to 1:400, we roughly matched the frequency of antigen specific B cells in the peripheral blood of immunized human donors, which is typically in the range of 0.1%–2% (Kodituwakku et al., 2003; Oshiba et al., 1994). The frequency of plasma cells recognizing leukemia-associated antigens (LAAs) in a cancer patient has not yet been determined, but their qualitative existence is well known in the literature (Tan, 2001; Smyth et al., 2001; Houghton, 1994), Even if present at a lower frequency, it should be possible to amplify them *in vitro* by stimulation with cytokines or ligands.

Another important aspect for the feasibility of patient screens is the number of target molecules on the cancer cells and hence the required sensitivity of the screening system. Routine immunohistochemistry (IHC) diagnostic tests have shown that the expression of Her2 antigen on the surface of breast cancer cells correlates with cancer progression and typically ranges from 5 to 23×10^5 molecules per cell. This is almost one order of magnitude more than the number of transferrin receptors on the surface of K562 cells used in this study (~1.5×10^5/cell) (Bridges and Smith, 1985; Lv et al., 2016; Ross et al., 2009).

Taken together, we believe our screening platform fulfills all requirements for the efficient screening of antibodies targeting membrane receptors or surface molecules involved in cancer or autoimmune diseases. This should open the way for many interesting screening approaches in the near future.

EXPERIMENTAL PROCEDURES

Flow Cytometric Analysis

For antibody binding assays, the K562 cells were stained with CTV (Thermo Fisher Scientific) dye and then fixed with 2% paraformaldehyde (PFA; Sigma). Cells were then treated with OKT 9 or H25B10 culture supernatants (1:100 and 1:500) or recombinant OKT 9 or H25B10 antibodies (50, 200, and 800 ng/mL) or CD55 (100, 400 and 1,600 ng/mL) or CD59 (400, 1,600 and 6,400 ng/mL) or CD3 (400, 1,600 and 6,400 ng/mL) or MUC1 (100, 400, and 1,600 ng/mL) antibodies. In all the samples Alexa-488-conjugated goat-anti-mouse antibody (2.5 μg/mL) was added. The cells were then analyzed in BD-LSRFortessa machine at EMBL Flow Cytometry Core Facility.

Determination of Viability of Hybridoma Cells after Droplet Encapsulation

OKT 9 and H25B10 hybridoma cells were washed 3 times with plain DMEM before encapsulation into droplets. Either OKT 9 or H25B10 cells were then injected into the droplet production chip as shown in Figure 1Bi, however, instead of K562 and fluorescently labeled antibodies, plain DMEM was injected. The aqueous phases were injected at a flow rate of 500 μL/hr, whereas Novec 7500 oil (Iolitec Liquids Technologies) with 1% PS-2 Surfactant (Sphere Fluidics) was injected at a flow rate of 4,000 μL/hr to produce droplets. After the cell encapsulation, the droplets were stored in the incubator at 37°C under a 5% CO_2 atmosphere. At various time intervals (2, 4, 6, 12, and 24 hr), 200 μL of emulsion was broken with an equal volume of 1H,1H,2H,2H-Perfluoro-1-octanol (PFO; Sigma), and cells were recovered from the aqueous phase. The recovered cells were then stained for 30–40 min with a staining solution containing Calcein-AM (2 μM, Thermo Fisher Scientific) and Ethidium Homodimer (4 μM, Sigma) in PBS. After incubation, images of the viable (green) and non-viable (red) cells were captured using a Nikon Ti-Eclipse microscope. The cells were counted within 4 different fields of view (>100 cells) for each time interval, from 3 independent experiments and plotted as mean viable cells ±SD.

Droplet Encapsulation of Cells/Beads, Droplet Sorting, and Imaging

All the cells were washed 3 times with plain DMEM (GIBCO) to remove FBS, before encapsulation. Before encapsulation, K562 cells were stained with CTV dye and fixed with 2% PFA (Sigma). For imaging experiments, OKT 9 cells were also labeled with CTFR dye (Thermo Fisher Scientific) as per manufacturer's instructions. Fluoresbrite blue-green microspheres (Polysciences) were washed 3 times with PBS, before encapsulation. The K562 cells (3×10^6/mL) and goat anti-mouse Alexa 488 antibodies (2.5 μg/mL) along with Xanthane gum (1 mg/mL; Sigma) were introduced from one inlet in the droplet generation chip at flow rate of 500 μL/hr. The OKT 9 and H25B10 cells (3×10^6/mL) along with Xanthane gum (1 mg/mL; Sigma) or purified antibodies, in case of recombinant antibody experiment, were introduced from another inlet in the droplet generation chip at flow rate of 500 μL/hr. Novec 7500 (Iolitec Liquids Technologies) with 1% PS-2 Surfactant (Sphere Fluidics) was used to produce droplets at the flow rate of 4,000 μL/hr. The droplets were collected and incubated for 1–2 hr. The emulsions were re-injected into the sorting chip using electro-osmotic pump (Nano Fusion Technologies) at a flow rate of about 100 μL/hr as described elsewhere (Hu et al., 2015). The FADS was performed using customized LabVIEW sorting program. Droplet imaging chip was connected with the outlet in the sorting chip to capture the sorted droplets (Figure 1Bii). The captured droplets were imaged using Zeiss Axiovert 200 as well as Nikon Ti Eclipse microscope.

Real-Time PCR Analysis of Sorted Cells

Cell were recovered from the sorted droplets by addition of 500 μL of plain DMEM followed by 300 μL of 1H,1H,2H,2H-Perfluoro-1-octanol (PFO; Sigma). After mixing thoroughly, the aqueous layer was carefully removed. The cells were then pelleted down and resuspended in 10 μL of lysis buffer containing 5% RNase inhibitor (Lucigen) and 0.5% Tween 20 (Sigma). The cells were further freeze-thawed 3 times. The cell lysate was then used for preparation of cDNA using oligodT primers and Superscript III cDNA synthesis kit (Thermo Fisher Scientific). Using the cDNA template, real-time PCR was set up with SYBR green PCR master mix (Thermo Fisher Scientific).

SUPPLEMENTAL INFORMATION

Supplemental Information includes Supplemental Experimental Procedures, seven figures, three tables, and two movies and can be found with this article online at https://doi.org/10.1016/j.celrep.2018.01.071.

ACKNOWLEDGMENTS

The authors gratefully acknowledge the funding received for this work from Worldwide Cancer Research (WCR 15-0187). Authors also acknowledge the technical support received from Diana Ordonez and Malte Paulsen (EMBL flow cytometry core facility) and from Vladimir Benes (EMBL Genomics core facility) and EMBL Advanced Light Microscopy Facility (ALMF). Authors would also like to thank Federica Eduati for her help in analyzing and plotting the fluorescence peak data.

AUTHOR CONTRIBUTIONS

C.A.M. conceived the project and conceptualized the normalization strategy. N.S. and C.A.M. designed experiments and analyzed data. N.S. performed all experiments except for the data shown in Figure 2. H.H. (experimental) and D.E. (software) established the normalization approach and obtained all data shown in Figure 2. N.S. and C.A.M. wrote the manuscript.

DECLARATION OF INTERESTS

C.A.M. is co-founder of Velabs Therapeutics. Parts of the work described in this study have been patented (EP15165915.8).

Received: July 28, 2017
Revised: December 18, 2017
Accepted: January 24, 2018
Published: February 20, 2018

REFERENCES

Bridges, K.R., and Smith, B.R. (1985). Discordance between transferrin receptor expression and susceptibility to lysis by natural killer cells. J. Clin. Invest. *76*, 913–918.

Chaipan, C., Pryszlak, A., Dean, H., Poignard, P., Benes, V., Griffiths, A.D., and Merten, C.A. (2017). Single-virus droplet microfluidics for high-throughput screening of neutralizing epitopes on HIV particles. Cell Chem. Biol. *24*, 751–757.

Chames, P., Van Regenmortel, M., Weiss, E., and Baty, D. (2009). Therapeutic antibodies: Successes, limitations and hopes for the future. Br. J. Pharmacol. *157*, 220–233.

Chao, G., Cochran, J.R., and Wittrup, K.D. (2004). Fine epitope mapping of anti-epidermal growth factor receptor antibodies through random mutagenesis and yeast surface display. J. Mol. Biol. *342*, 539–550.

Clausell-Tormos, J., Lieber, D., Baret, J.C., El-Harrak, A., Miller, O.J., Frenz, L., Blouwolff, J., Humphry, K.J., Köster, S., Duan, H., et al. (2008). Droplet-based microfluidic platforms for the encapsulation and screening of Mammalian cells and multicellular organisms. Chem. Biol. *15*, 427–437.

Ecker, D.M., Jones, S.D., and Levine, H.L. (2015). The therapeutic monoclonal antibody market. MAbs *7*, 9–14.

El Debs, B., Utharala, R., Balyasnikova, I.V., Griffiths, A.D., and Merten, C.A. (2012). Functional single-cell hybridoma screening using droplet-based microfluidics. Proc. Natl. Acad. Sci. USA *109*, 11570–11575.

Eyer, K., Doineau, R.C.L., Castrillon, C.E., Briseño-Roa, L., Menrath, V., Mottet, G., England, P., Godina, A., Brient-Litzler, E., Nizak, C., et al. (2017). Single-cell deep phenotyping of IgG-secreting cells for high-resolution immune monitoring. Nat. Biotechnol. *35*, 977–982.

Houghton, A.N. (1994). Cancer antigens: Immune recognition of self and altered self. J. Exp. Med. *180*, 1–4.

Hu, H., Eustace, D., and Merten, C.A. (2015). Efficient cell pairing in droplets using dual-color sorting. Lab Chip *15*, 3989–3993.

Huang, J., Doria-Rose, N.A., Longo, N.S., Laub, L., Lin, C.L., Turk, E., Kang, B.H., Migueles, S.A., Bailer, R.T., Mascola, J.R., and Connors, M. (2013). Isolation of human monoclonal antibodies from peripheral blood B cells. Nat. Protoc. *8*, 1907–1915.

Hutchings, C.J., Koglin, M., and Marshall, F.H. (2010). Therapeutic antibodies directed at G protein-coupled receptors. MAbs *2*, 594–606.

Joensson, H.N., Samuels, M.L., Brouzes, E.R., Medkova, M., Uhlén, M., Link, D.R., and Andersson-Svahn, H. (2009). Detection and analysis of low-abundance cell-surface biomarkers using enzymatic amplification in microfluidic droplets. Angew. Chem. Int. Ed. Engl. *48*, 2518–2521.

Kodituwakku, A.P., Jessup, C., Zola, H., and Roberton, D.M. (2003). Isolation of antigen-specific B cells. Immunol. Cell Biol. *81*, 163–170.

Konry, T., Dominguez-Villar, M., Baecher-Allan, C., Hafler, D.A., and Yarmush, M.L. (2011). Droplet-based microfluidic platforms for single T cell secretion analysis of IL-10 cytokine. Biosens. Bioelectron. *26*, 2707–2710.

Lv, Q., Meng, Z., Yu, Y., Jiang, F., Guan, D., Liang, C., Zhou, J., Lu, A., and Zhang, G. (2016). Molecular mechanisms and translational therapies for human epidermal receptor 2 positive breast cancer. Int. J. Mol. Sci. Published online December 14, 2016. https://doi.org/10.3390/ijms17122095.

Mazutis, L., Gilbert, J., Ung, W.L., Weitz, D.A., Griffiths, A.D., and Heyman, J.A. (2013). Single-cell analysis and sorting using droplet-based microfluidics. Nat. Protoc. *8*, 870–891.

Midgett, C.R., and Madden, D.R. (2007). Breaking the bottleneck: Eukaryotic membrane protein expression for high-resolution structural studies. J. Struct. Biol. *160*, 265–274.

Nelson, A.L., Dhimolea, E., and Reichert, J.M. (2010). Development trends for human monoclonal antibody therapeutics. Nat. Rev. Drug Discov. *9*, 767–774.

Oshiba, A., Renz, H., Yata, J., and Gelfand, E.W. (1994). Isolation and characterization of human antigen-specific B lymphocytes. Clin. Immunol. Immunopathol. *72*, 342–349.

Reichert, J.M. (2012). Marketed therapeutic antibodies compendium. MAbs *4*, 413–415.

Reichert, J.M. (2016). Antibodies to watch in 2016. MAbs *8*, 197–204.

Reichert, J.M. (2017). Antibodies to watch in 2017. MAbs *9*, 167–181.

Ross, J.S., Slodkowska, E.A., Symmans, W.F., Pusztai, L., Ravdin, P.M., and Hortobagyi, G.N. (2009). The HER-2 receptor and breast cancer: Ten years of targeted anti-HER-2 therapy and personalized medicine. Oncologist *14*, 320–368.

Ryall, R.G., Story, C.J., and Turner, D.R. (1982). Reappraisal of the causes of the "hook effect" in two-site immunoradiometric assays. Anal. Biochem. *127*, 308–315.

Scott, A.M., Wolchok, J.D., and Old, L.J. (2012). Antibody therapy of cancer. Nat. Rev. Cancer *12*, 278–287.

Shembekar, N., Chaipan, C., Utharala, R., and Merten, C.A. (2016). Droplet-based microfluidics in drug discovery, transcriptomics and high-throughput molecular genetics. Lab Chip *16*, 1314–1331.

Smyth, M.J., Godfrey, D.I., and Trapani, J.A. (2001). A fresh look at tumor immunosurveillance and immunotherapy. Nat. Immunol. *2*, 293–299.

Staudt, N., Müller-Sienerth, N., and Wright, G.J. (2014). Development of an antigen microarray for high throughput monoclonal antibody selection. Biochem. Biophys. Res. Commun. *445*, 785–790.

Sutherland, R., Delia, D., Schneider, C., Newman, R., Kemshead, J., and Greaves, M. (1981). Ubiquitous cell-surface glycoprotein on tumor cells is proliferation-associated receptor for transferrin. Proc. Natl. Acad. Sci. USA *78*, 4515–4519.

Tan, E.M. (2001). Autoantibodies as reporters identifying aberrant cellular mechanisms in tumorigenesis. J. Clin. Invest. *108*, 1411–1415.

Weiner, G.J. (2015). Building better monoclonal antibody-based therapeutics. Nat. Rev. Cancer *15*, 361–370.

Cell Reports, Volume *22*

Supplemental Information

Single-Cell Droplet Microfluidic Screening for Antibodies Specifically Binding to Target Cells

Nachiket Shembekar, Hongxing Hu, David Eustace, and Christoph A. Merten

Supplemental information

A] Supplemental Figures & Tables

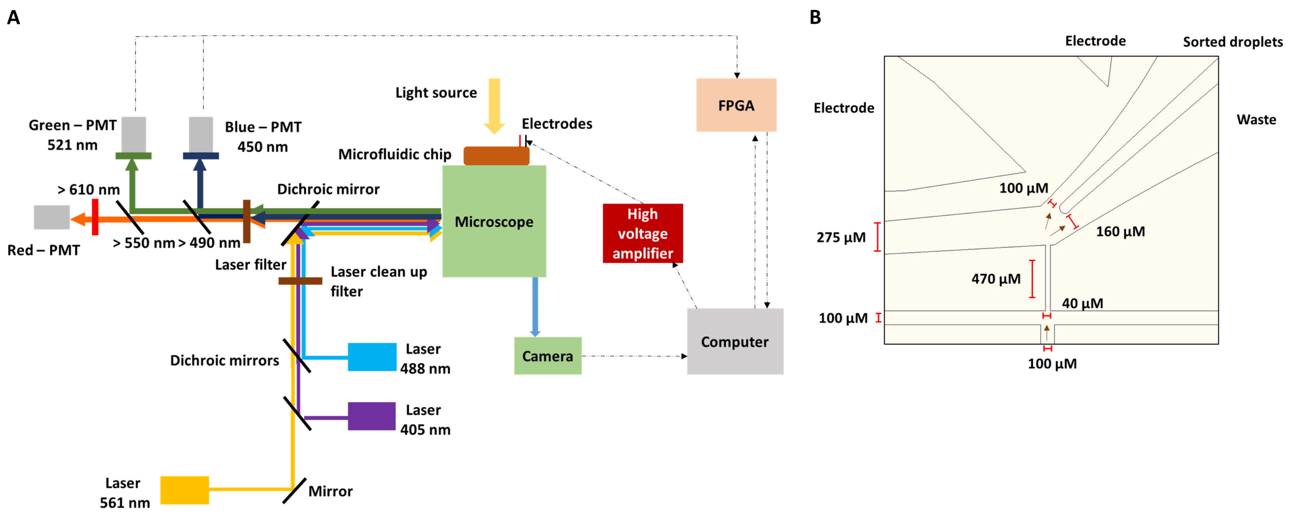

Figure S1: Optical setup and geometry of the sorting junction, Related to Figure 1

[A] The droplets moving in the microfluidic chip were excited with lasers having excitation wavelengths of 405 nm (CTV dye) and 488 nm (Alexa488-goat-anti-mouse IgG antibodies), while the 561 nm laser is an additional optional that was not used in our assays. The emission signals were detected using photomultiplier tubes (PMTs) with a 450 nm band-pass filter (blue), a 521 nm band-pass filter (green), and a 610 nm longpass filter (red). Droplet sorting signals were processed using a LabVIEW software program running on a FPGA card triggering a high voltage amplifier. Imaging was performed using an inverted microscope equipped with a high speed camera. [B] Dimensions of the sorting junction of the microfluidic device used for droplet sorting [Fig. 1B (ii)]. Brown arrows represent the direction in which the droplets move in the sorting device.

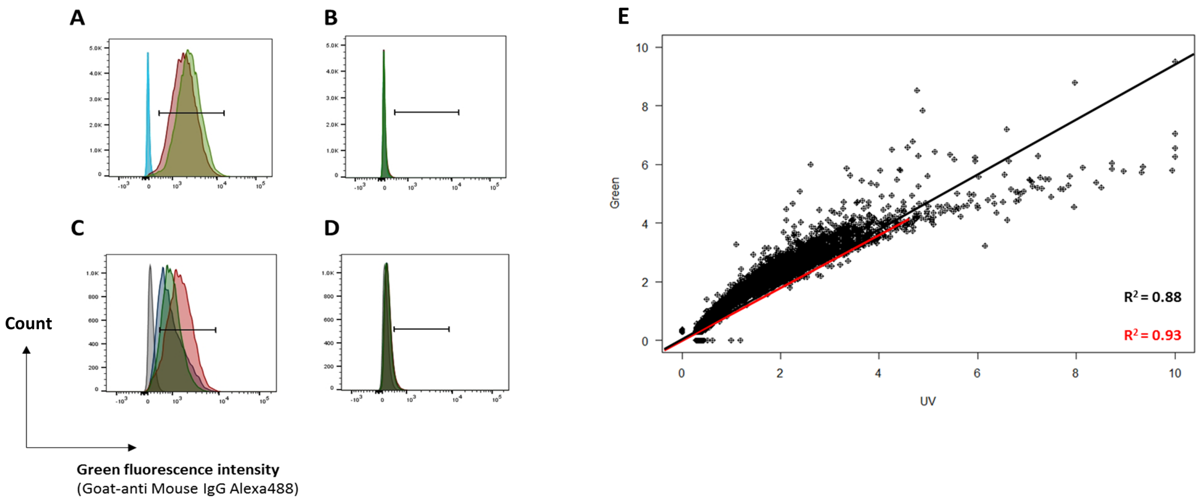

Figure S2: Flow cytometric detection of OKT 9 antibody binding and correlation of normalized signal intensities, Related to Figure 2 and 3

K562 cells were treated with 1:100 (red) and 1:500 (green) dilutions of culture supernatant of OKT 9 [A] or H25B10 [B] cells. The OKT 9 cell secreted antibodies [A] showed significant binding to K562 cells; whereas H25B10 cell secreted antibodies [B] failed to show any binding, as compared to the control (cyan). Further, K562 cells were probed with recombinant OKT 9 [C] or H25B10 [D] antibodies at 50 (blue), 200 (red) and 800 ng/ml (green) concentrations. The OKT 9 antibodies [C] showed binding to the K562 cells whereas H25B10 antibodies [D] did not show any binding, as compared to the control sample (grey). Due to competition for limited secondary antibody between antigen-bound and excessive soluble primary antibody, the OKT 9 binding to K562 cells [C] at 800 ng/ml (green) showed a slightly weaker signal as compared to 200 ng/ml (red). [E] The analysis of significance of correlation between normalized green vs. blue intensity (Fig. 2) showed that >80% of the population shows significant correlation with an R^2-value of 0.93 up to a cut-off value of 4 (shown in red); as compared to an R^2-value of 0.88 (shown in black) for the entire population.

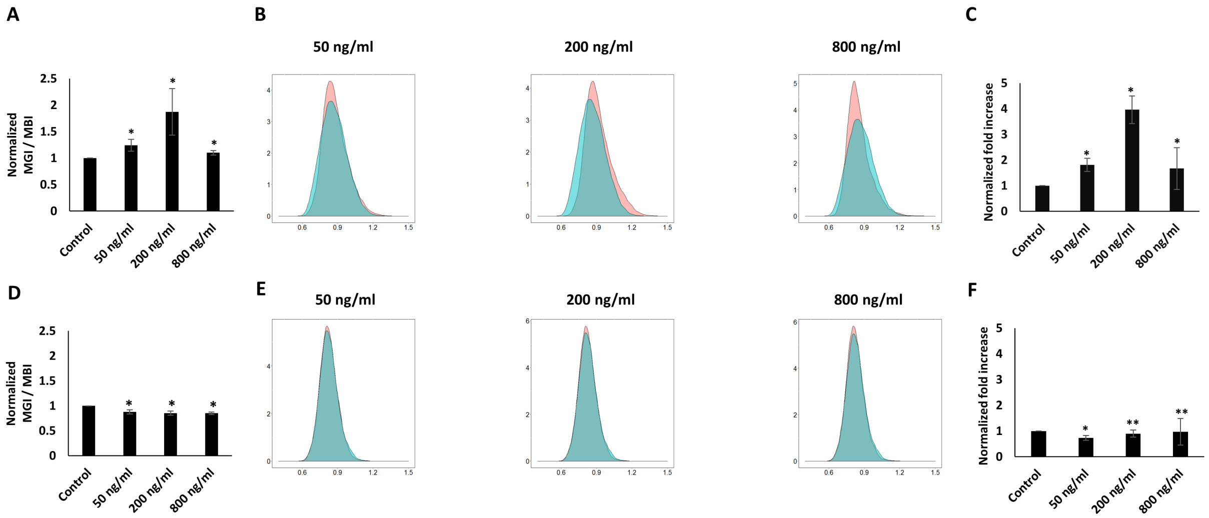

Figure S3: Comparative analysis of relative mean and individual fluorescence intensities from recombinant antibody binding droplet data, Related to Figure 3

To compare the relative increase in fluorescence intensities after recombinant [A] OKT 9 antibody binding on the target cell in droplets, the normalized ratio of Mean Green fluorescence Intensity (MGI) against Mean Blue fluorescence Intensity (MBI) was plotted. It was observed that the [A] OKT 9 antibody binding to target cells lead to relatively higher green fluorescence intensity for 50 – 800 ng/ml antibody as compared to the control (absence of primary antibody). [B] To analyse the robustness of the antibody binding assay, fluorescence intensities after recombinant [B] OKT 9 binding on the K562 cells in droplets were plotted as the ratio of green fluorescence intensity against blue fluorescence intensity for each individual data point shown in Fig. 3A (40,000 – 60,000 peaks in total). [C] Fold increase in the number of data points with a green / blue ratio above 1.1 for OKT 9 over the control (normalized to 1). [D] In contrast, the ratio of MGI / MBI for H25B10 antibody even showed slightly decreased green fluorescence intensity for 50 – 800 ng/ml antibody concentration as compared to the control, potentially indicating a higher level of unspecific binding of the secondary antibody than that of the H25B10 antibody. [E] Similarly, fluorescence intensities after recombinant H25B10 binding on the K562 cells in droplets were plotted as the ratio of green fluorescence intensity against blue fluorescence Intensity for each individual data point shown in Fig. 3B and [F] fold increase in the number of data points with a green / blue ratio above 1.1. Averaged data of 3 independent experiments is shown. The values have been expressed as Mean $\pm$ SD. *$p < 0.05$, **$p > 0.05$ as compared to the control.

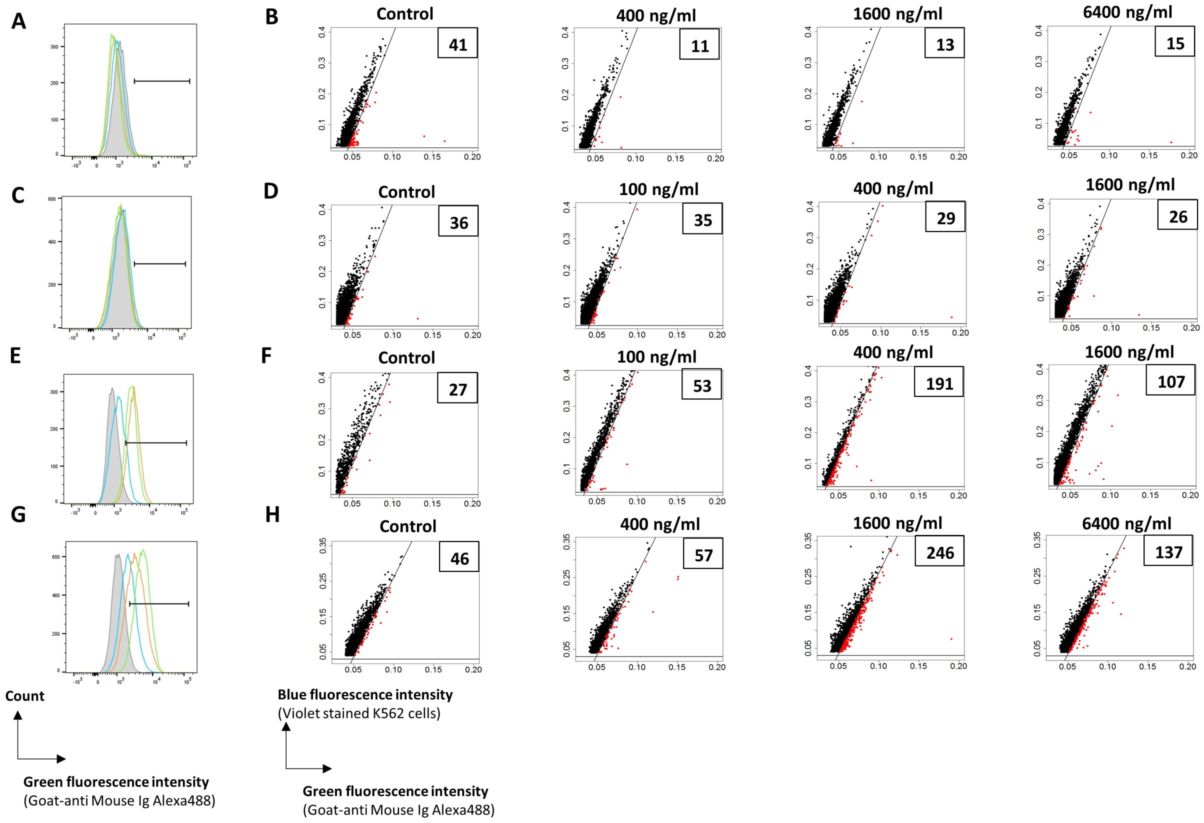

Figure S4: Flow cytometric and droplet based analysis of CD3, MUC1, CD55 and CD59 antibody binding on K562 cell surface, Related to Figure 3

K562 cells treated with recombinant purified CD3 antibody (400-1600-6400 ng/ml; [A] Cyan-Orange-Green, respectively) neither showed any specific antibody binding over the control (filled grey) in flow-cytometric analysis [A] nor in droplets [B] based analysis of fluorescent peaks. Similarly, the K562 cells treated with recombinant purified MUC1 antibody (100-400-1600 ng/ml; [C] Cyan-Orange-Green, respectively) neither showed any specific antibody binding over the control (filled grey) in flow-cytometric analysis [C] nor in droplet [D]. K562 cells treated with recombinant purified CD55 antibody (100-400-1600 ng/ml; Cyan-Orange-Green, respectively) [E] showed significant antibody binding over the control (filled grey) in flow-cytometric analysis. [F] Similar analysis of fluorescent peaks in a droplet based system using CD55 antibody showed significant antibody binding events (53-191-107), over the control sample (27) that did not contain any primary antibody. K562 cells treated with recombinant purified CD59 antibody (400-1600-6400 ng/ml; Cyan-Orange-Green, respectively) [G] showed significant antibody binding over the control (filled grey) in flow-cytometric analysis. [H] Similarly, analysis of fluorescent peaks in a droplet based system using CD59 antibody showed significant antibody binding events (57-246-137), over the control sample (46) that did not contain any primary antibody. Fluorescence peak intensities from 20,000 droplets have been plotted along with horizontal and diagonal lines mimicking a sorting gate [B, D, F and H]. Peaks in the sorting gate have been shown in red color as well as represented in numbers inside the box.

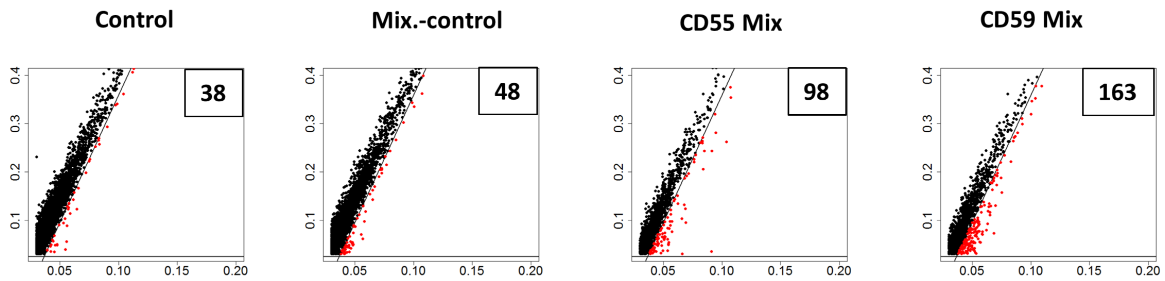

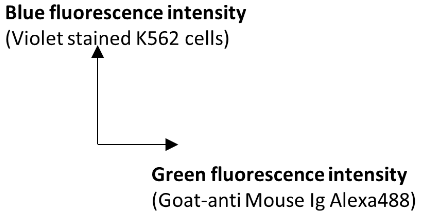

	Control	Mix. –Control	CD55 Mix	CD59 Mix
CD55			√	
CD59				√
H25B10		√	√	√
CD3		√	√	√
MUC1		√	√	√
Anti-Mouse Ig Alexa488	√	√	√	√

Figure S5: Droplet based analysis of CD55 and CD59 antibody binding on K562 cell surface in presence of non-specific antibodies, Related to Figure 4

CTV stained K562 cells along with anti-mouse-Ig-Alexa488 were probed in the droplets either with plain medium (Control) or non-specific antibody mixtures (Mix.-control) or antibody mixtures containing either CD55 or CD59 antibody (CD55 Mix or CD59 Mix) (1600 ng/ml) (see table for details; specific binders are highlighted in green and unspecific antibodies are highlighted in red). The fluorescence peak data obtained from the droplets (20,000 peaks) was plotted as green vs. blue fluorescence intensity. The diagonal and horizontal lines have been drawn so as to mimic a sorting gate to sort droplets showing relatively higher green fluorescence intensity, also represented in red colour and numbered in the box. The presence of CD55 or CD59 antibody even in a mixture of different non-specific antibodies resulted in significantly increased numbers of peaks with relatively higher green fluorescence intensity over the control samples.

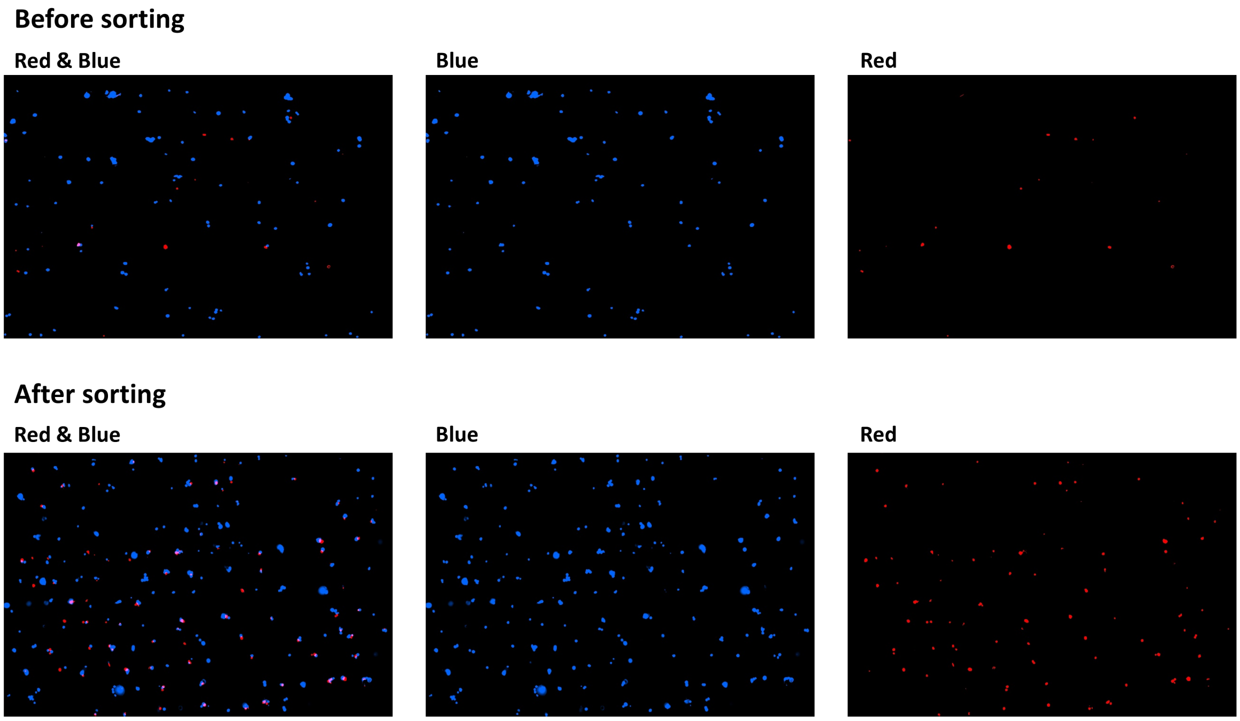

Figure S6: Analysis of a larger population of droplets before and after sorting, Related to Figure 6

To analyse the enrichment of correct droplets after FADS, the droplets before and after sorting were imaged. The K562 cells (blue) and OKT 9 cells (red) were labelled with different dyes and each image is shown in red and blue, blue only and red only channels.

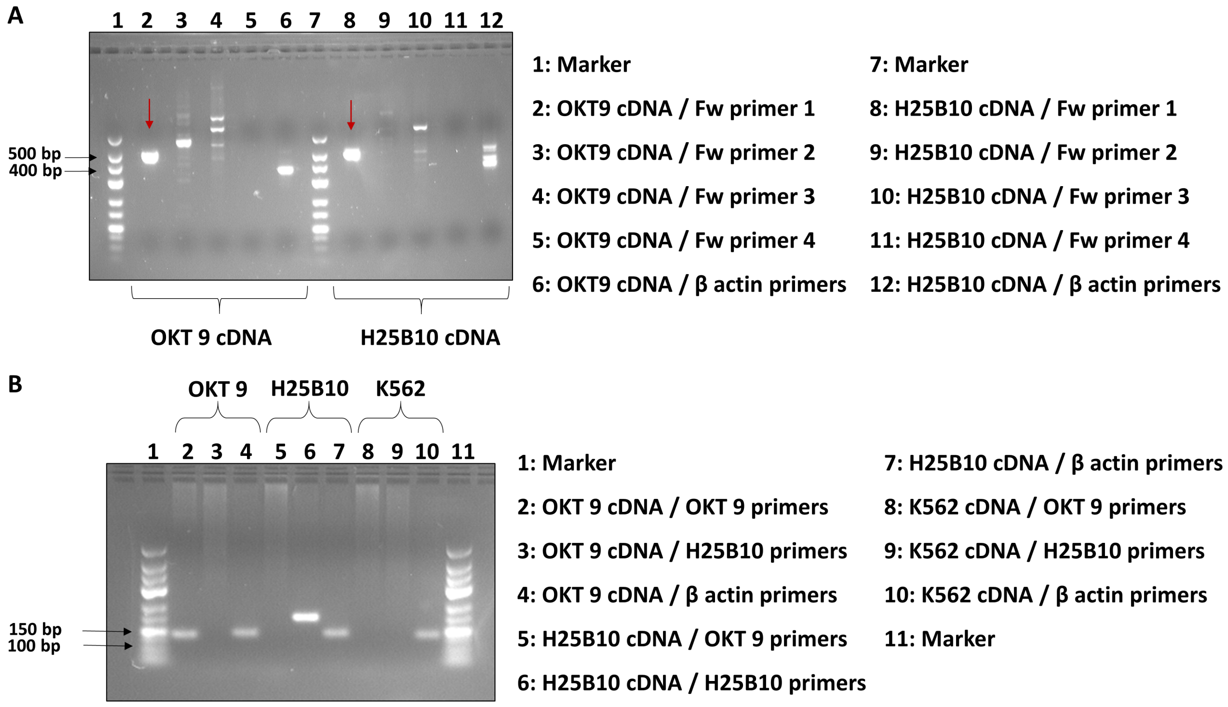

Figure S7: Amplification of OKT 9, H25B10 heavy chain antibody V regions for sequencing and designing of specific Real Time PCR primers, Related to Figure 7

[A] In order to sequence the heavy chain variable (V) region of OKT 9 and H25B10 antibodies, the cDNA from respective hybridoma cells were amplified with universal PCR primers; each in combination with 4 forward (Fw) and 1 reverse primers to amplify the unknown V regions. The amplified products were resolved on 2% agarose gel and products with appropriate size (~450 bp), also shown by red arrows, were further sent for sequencing. Beta-actin primers were used as a non-quantitative internal positive control for the PCR. [B] Real time PCR primers were designed to specifically amplify a sequence (~ 100 bp) within the heavy chain V region of OKT 9 (2, 3) and H25B10 (5, 6) antibodies. In addition, β-actin primers were also designed to amplify beta actin gene (~100 bp) in OKT 9 (4), H25B10 (7) and K562 (10) cell lines, for normalization of real time PCR data. All the PCR products were resolved on 2% agarose gel. All the primers were designed to have similar Tm (48^0 C) and amplification efficiency.

Table S1: Primers for amplification of OKT9 and H25B10 antibody heavy chain V region, Related to Figure 7

No.	Primer	Sequence
1.	Mouse Antibody Heavy chain reverse	5 ′- AGGTCTAGAAYCTCCACACACAGGRRCCAGTGGATAGAC - 3′
2.	Mouse Antibody Heavy chain forward 1	5′- GGGGATATCCACCATGGRATGSAGCTGKGTMATSCTCTT- 3′
3.	Mouse Antibody Heavy chain forward 2	5′-GGGGATATCCACCATGRACTTCGGGYTGAGCTKGGTTTT-3′
4.	Mouse Antibody Heavy chain forward 3	5′-GGGGATATCCACCATGGCTGTCTTGGGGCTGCTCTTCT-3′
5.	Mouse Antibody Heavy chain forward 4	5′-GGGGATATCCACCATGGRCAGRCTTACWTYY-3′

Table S2: Real time PCR primers for specific amplification of Beta actin, OKT 9 and H25B10 antibody V regions, Related to Figure 7

No.	Primer	Sequence
1.	OKT 9 forward	5’ – CTGTCAGGAACTGCAGGT – 3’
2.	OKT 9 reverse	5’ – GGAAATATTCATTGAAGT – 3’
3.	H25B10 forward	5’ – CCTAGCAACGGTCGTTCC – 3’
4.	H25B10 reverse	5’ – AAGGTTACCTACTCTTGC – 3’
5.	Beta-actin forward	5’ – TGTATGAAGGCTTTGGTCTCC – 3’
6.	Beta-actin reverse	5’ – TGTGCACTTTTATTGGTCTCAAG – 3’

Table S3: Real time PCR standard curve for analysis of enrichment of OKT 9 cells, Related to Figure 7

No. of K562 Cells	No. of OKT 9 cells	No. of H25B10 cells	Ct value for OKT 9 amplification	Ct value for H25B10 amplification	Ratio of Ct values (OKT 9 / H25B10)	% OKT 9 cells enrichment
50	50	-	26.05	30.2	0.86	100
50	40	10	26.88	30.1	0.89	80
50	25	25	30.24	28.37	1.10	50
50	10	40	31.93	27.45	1.20	20
50	-	50	32.99	26.58	1.24	0

B] Supplemental experimental procedures

Cell lines, antibodies and dyes

The K562 (ATCC® CCL-243™), OKT 9 hybridoma (ATCC® CRL-8021™) and H25B10 hybridoma (ATCC® CRL-8017A™) cell lines were purchased from ATCC. The K562 cells were cultured in Iscove's Modified Dulbecco's Medium (IMDM) (ATCC® 30-2005™) with 10% FBS (Gibco); whereas OKT 9 and H25B10 hybridoma cells were cultured in IMDM with 20% FBS. Recombinant OKT9 mouse monoclonal antibody (Anti-human CD71, Transferrin receptor) was purchased from affymetrix eBioscience. Recombinant H25B10 monoclonal antibody (Mouse anti-Hepatitis B virus surface antigen) was procured from Merck Millipore. Mouse anti-human-CD55 antibody (referred to as "CD55"), mouse anti-human-CD59 antibody (referred to as "CD59") and mouse anti-human-CD3 antibody (referred to as "CD3") were purchased from Biolegend, USA. Mouse-anti-human MUC1 antibody (referred to as "MUC1") was obtained from Abcam. Goat-anti-mouse IgG Alexa fluor 488 conjugated antibody, CellTrace Violet and CellTrace Far Red dyes were purchased from Thermo Fisher scientific.

Design and fabrication of microfluidic devices

All the microfluidic devices were designed in AutoCAD and fabricated using soft lithography as described previously (Hu et al., 2015). Briefly, silicon wafers (Silicon Materials) were coated with SU-8 photoresist (Microchem) and patterned by UV-illumination through high resolution lithography masks (Selba). Microfluidic chips were prepared by pouring a mixture of 9:1 (w/w) Polydimethylsiloxane (PDMS) elastomer (Sylgard 184 polymer base; Dow Corning) and curing agent (Dow Corning) over the molds which were then cured overnight at 65^{0} C. Polymerized PDMS was peeled off from the molds and inlets for tubings and electrodes were punched by biopsy punches (Acuderm inc.). Subsequently the PDMS-devices were plasma bonded onto microscopy glass slides (Thermo Fisher Scientific Inc.; chips for droplet production and capture) or onto ITO glass (Delta Technologies LTD; chips for sorting). Finally, the channels were made hydrophobic by treatment with Aquapel.

Analysis of fluorescence peak data

The fluorescence peak data obtained from PMT was analysed and plotted using the R-software.

Amplification and sequencing of OKT 9 and H25B10 antibody V region

Total RNA was isolated from OKT 9 and H25B10 hybridoma cells using RNeasy Mini kit (Qiagen). The RNA was then converted to cDNA using Superscript III one step RT PCR with platinum Hi-Fi Taq (Thermo Fisher) employing combination of specific reverse and forward primers for heavy chain V region [Table S1] (Morrison, 2002). The PCR products were resolved on 2 % agarose (Sigma) gel. The products with appropriate size (~450 bp) were eluted out from the gel and sent for sequencing (GATC Biotech).

Re-cultivation of cells and second round of FADS

After the first round of droplet sorting, positively sorted droplets (500 – 1,000) were broken and cells were recovered as described earlier. The recovered cells were then cultured in a 96-well plate in medium containing equal amounts of fresh IMDM-20% FBS and (OKT 9 or H25B10-) conditioned medium for 2 weeks. Subsequently, cells were gradually transferred to 6-well plates and finally to T25 culture flasks. The second round of FADS was performed as described earlier, using equal amounts of K562 cells and re-cultivated hybridoma cells along with the fluorescently labelled goat-anti-Mouse Ig-Alexa488. After the sorting, the cells were again recovered from the sorted droplets and processed for real time PCR analysis, as described earlier.

Supplemental References

HU, H., EUSTACE, D. & MERTEN, C. A. 2015. Efficient cell pairing in droplets using dual-color sorting. *Lab Chip,* 15**,** 3989-93.

MORRISON, S. L. 2002. Cloning, expression, and modification of antibody V regions. *Curr Protoc Immunol,* Chapter 2**,** Unit 2 12.
